# Eugene – A Domain Specific Language for Specifying and Constraining Synthetic Biological Parts, Devices, and Systems

**DOI:** 10.1371/journal.pone.0018882

**Published:** 2011-04-29

**Authors:** Lesia Bilitchenko, Adam Liu, Sherine Cheung, Emma Weeding, Bing Xia, Mariana Leguia, J. Christopher Anderson, Douglas Densmore

**Affiliations:** 1 Department of Computer Science, California State Polytechnic University, Pomona, California, United States of America; 2 Department of Electrical Engineering and Computer Sciences, University of California, Berkeley, California, United States of America; 3 Department of Bioengineering, University of California San Diego, La Jolla, California, United States of America; 4 Department of Chemical Engineering and Materials Science, University of Minnesota, Minneapolis, Minnesota, United States of America; 5 Department of Bioengineering, QB3: California Institute for Quantitative Biological Research, University of California, Berkeley, California, United States of America; 6 Physical Biosciences Division, Lawrence Berkeley National Laboratory, Berkeley, California, United States of America; 7 Department of Electrical and Computer Engineering, Boston University, Boston, Massachusetts, United States of America; Fondazione Telethon, Italy

## Abstract

**Background:**

Synthetic biological systems are currently created by an ad-hoc, iterative process of specification, design, and assembly. These systems would greatly benefit from a more formalized and rigorous specification of the desired system components as well as constraints on their composition. Therefore, the creation of robust and efficient design flows and tools is imperative. We present a human readable language (Eugene) that allows for the specification of synthetic biological designs based on biological parts, as well as provides a very expressive constraint system to drive the automatic creation of composite Parts (Devices) from a collection of individual Parts.

**Results:**

We illustrate Eugene's capabilities in three different areas: Device specification, design space exploration, and assembly and simulation integration. These results highlight Eugene's ability to create combinatorial design spaces and prune these spaces for simulation or physical assembly. Eugene creates functional designs quickly and cost-effectively.

**Conclusions:**

Eugene is intended for forward engineering of DNA-based devices, and through its data types and execution semantics, reflects the desired abstraction hierarchy in synthetic biology. Eugene provides a powerful constraint system which can be used to drive the creation of new devices at runtime. It accomplishes all of this while being part of a larger tool chain which includes support for design, simulation, and physical device assembly.

## Introduction

In its development as an engineering field, *synthetic biology* is at a stage where encapsulation has been identified as a fundamental challenge [1], [2], [3], [4]. Encapsulation will enable design re-use, sharing, and software tool development, all of which greatly increase synthetic biology's ability to grow both in complexity and in community size. Encapsulation has been shown to be very important in other engineering disciplines [5], [6], [7]. We present a domain specific programming language called Eugene meant to encapsulate biological *Parts*, *Devices*, and *Rules* paving the way for *design space exploration*, *simulation*, and *automated assembly*.

**Figure 1:**
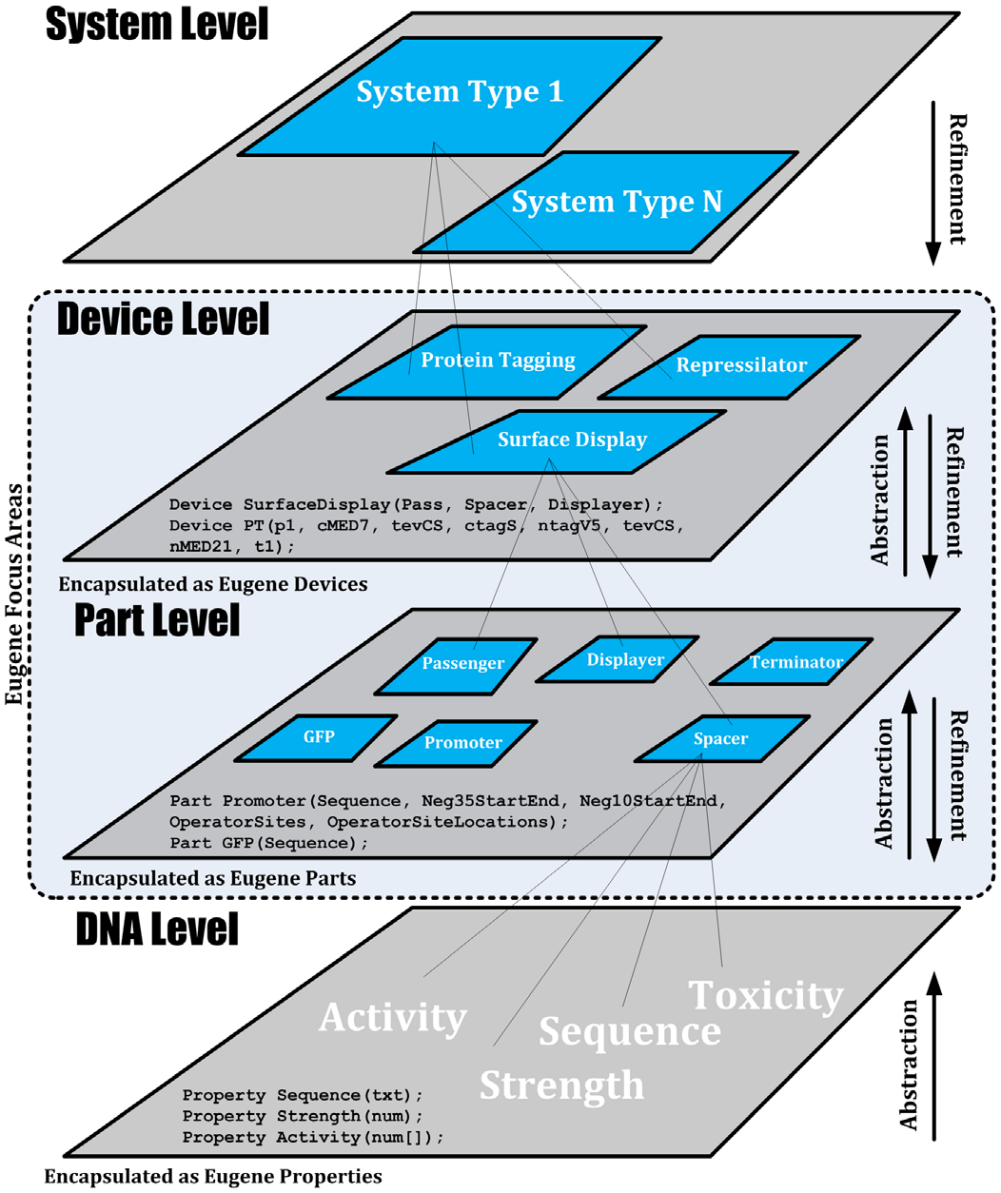
Encapsulation based synthetic biology design hierarchy. Shown are the various layers of abstraction at which Eugene operates. DNA information forms the most basic unit on which everything else is built (e.g. the genetic code, as specified by bases G, A, T, and C). This is followed by Parts. Parts are non-reducible elements of genetic composition (e.g. promoters, ribosome binding sites, open reading frames, etc). Devices, which can contain one or more Parts, are the next level in the hierarchy. Finally, Devices are followed by a *System* view that contains collections of Devices. The traversal upward in the hierarchy represents an abstraction process while a downward traversal represents the refinement process. Eugene currently operates at the Part and Device levels via explicit Part and Device data types while encapsulating the DNA level as Eugene Properties.

One popular encapsulation view in synthetic biology is that DNA sequence information can be encapsulated as a *Part*. Parts are well defined regarding the way in which they can be physically composed to create *Devices* [8], [9]. Parts and Devices then can be re-used in various designs, thus encouraging the development of new larger constructs for the community (see Figure 1) [10]. The process of developing standardized and well-characterized Parts is a key challenge, and community efforts in this direction have been undertaken through the BioBricks Foundation™ (http://bbf.openwetware.org/), the OpenWetWare initiative (http://openwetware.org/), and the International Genetically Engineered Machine (iGEM) competition (http://www.igem.org) [11].

Eugene (a play on the Greek prefix “*eu*” meaning “good” and the word “gene”) is a human readable, executable specification [12], which reflects the creation of systems by defining, specifying, and combining collections of Parts. Eugene is inspired by the languages of the Electronic Design Automation (EDA) [13], [14] industry (e.g. Verilog [15] and VHDL [16]) in terms of its ability to provide a biological design *netlist* (a collection of components and their connections). This can be *synthesized* (automatically transformed) into collections of physical implementations in a design library [17].

**Figure 2:**
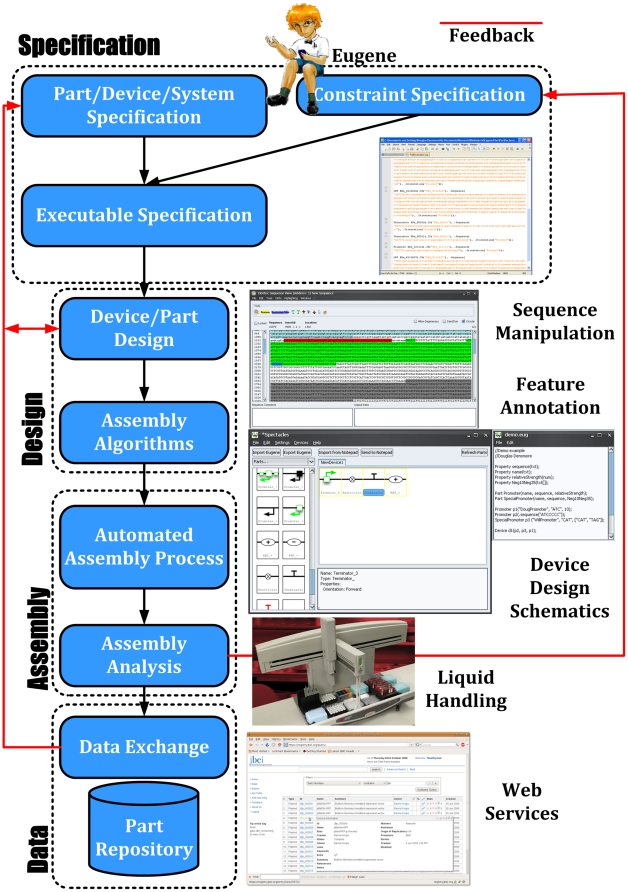
Eugene based synthetic biology design flow. Shown here is the role that Specification, Design, Assembly, and Data can play in synthetic biology. In particular, we illustrate that Eugene is concerned with the activities at the specification level explicitly but at the same time it is designed in such a way that it develops designs that are amenable to other pieces of this design flow. Opportunities for the flow to provide feedback to earlier stages and perform iterative refinement are outlined in red.

Eugene development has focused on:

1. Flexible Part and Device specification and composition (see Methods and Supplemental Information).
2. Combinatorial design space exploration of Devices using an expressive system of Rules [18] (see Results).
3. Interaction with other tools for simulation and automated assembly (see Results and Figure 2).

**Figure 3:**
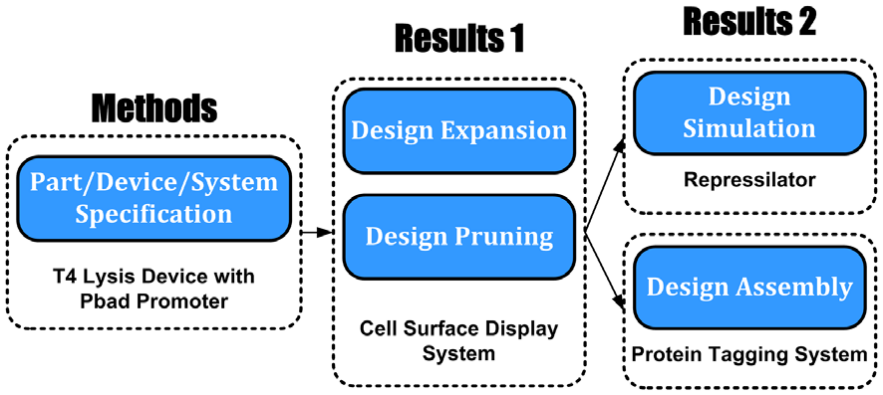
This paper is organized around three sections which reflect a Eugene design flow. The Methods section provides an overview of how to use Eugene to create a Device using a T4 lysis Device from the MIT parts registry. The Results section illustrates design space exploration with a *Cell Surface Display* system from UC Berkeley's 2009 iGEM team. The Results section also details integration of Eugene with automated assembly in Clotho for a protein tagging system and simulation via SynBioSS for a repressilator design.

This paper is organized around these three areas as shown in Figure 3.

## Methods

### Device Specification

Eugene is composed of *primitives*, *constructs*, *rules*, and *functions*. These elements are outlined in Table 1 along with a brief explanation. For the sake of brevity, we cannot cover this material in depth. For more details, see the Supplemental Information, http://www.eugenecad.org, and [19], which are devoted to covering Eugene's inner workings.

**Table 1:** Eugene language elements overview.

Primitives		
Name	Description	Examples
txt	txt variables store strings of zero or more characters.	txt message = "hello world";txt sequence = "ACTG";
num	num variables store numbers, both integer and floating point	num num1, num2;num1 = 1.0;
boolean	boolean values are either true or false.	boolean flag = true;
txt[] and num[]	txt[] and num[] are arrays of the corresponding singleton primitives.	txt[] str_array = ["A", "CG", "T"];num[] num_array = [1], [2], [3], [4];num num1 = num_array[0];
**Properties**		
Property Definition	Property definitions assign names to possible properties of Parts. Properties have a name and map to a primitive type.	Property ID(num);Property sequence(txt);Property strength(num);
**Parts**		
Part Definition	Part definitions define the fields of a type of Part. The fields are defined by using pre-defined properties.	Part Promoter(ID, sequence, strength);Part CDS(ID, sequence);
Part Declaration	Once a part type is defined, instances of that Part type can be declared, initialized, and used.	Promoter P1(1, "TATATA", 30);CDS GFP(1, “ATG…”);
**Devices**		
Device Declaration	Devices represent a composite Part. They can include both parts and other Devices as subcomponents. Devices are ordered 5′ to 3′.	Device BBa_1(P1, GFP);BBa_1[0]; //References P1
**Rules**		
Rule Declaration	Rule declarations use rule operators (such as BEFORE and CONTAINS) to describe constraints on Devices. Rules need to be declared before they can be used.	Rule R1(P1 BEFORE GFP);Rule R2(BBa_1 CONTAINS P1);
Assert and Note	Once declared, rules can either be asserted or noted. Asserted rules throw an exception when the rule is not satisfied, while noted rules print an error message.	Assert(R1);Note(R2);
**Header Files**		
Header files containing predefined properties, Part definitions, and Part declarations in separate files can be imported into a Eugene file.		
**Utility Functions**		
print()	A simple print function to allow output of data.	print("hello world");print(P1);
permute()	Permute creates all the permutations of a given Device. This is done by swapping out each component part of a Device with other instances of the same Part type.	Device BBa1(P1, GFP);permute(BBa1);BBa1_2; //Accesses the 2nd permutation

A short summary of the Eugene language specification is provided. While not a complete explanation, this table highlights key features and shows how the elements are organized. A complete description of both the language and technology used to implement it can be found at http://www.eugenecad.org. Further examples can be found in the Supplemental Information.

To provide the reader with the required understanding of Eugene, we will step through the creation of a “T4 Lysis Device with Pbad as the inducible Promoter”. This is a *standardized biological part* and can be retrieved as *BBa_K112809* in the MIT Registry of Standard Biological Parts (http://partsregistry.org).

Before beginning, it should be pointed out that there are two approaches to design in Eugene:

1. **Bottom-Up Design (BUD)** – BUD begins with low-level Properties, creates individual Parts, and then creates Devices. BUD is how libraries of Parts in Eugene will be created but requires a very detailed understanding of the system being created *a priori*.
2. **Top-Down Design (TDD)** – TDD begins by specifying the Devices of interest and then instantiating Parts, and finally specifying the Properties that make up the Parts. TDD is a very natural way to design systems, but in the absence of the lower- level elements the design is incomplete.

Our example follows the TDD paradigm in the interest of clarity.

#### Step 1: Specify the Header Files

These files encapsulate information on libraries of Eugene design elements at your disposal. Eugene comes with pre-created sample Header Files. Users can create their own Header Files manually or automatically (more in the Supplemental Information). Here the Header Files are divided into categories detailing what they contain. This separation is not a requirement.

include PropertyDefinition.h, PartDefinition.h, PartDeclaration.h;

#### Step 2: Specify the Device(s)

Devices are collections of 1) Parts or 2) other Devices. These must be specified in the body of the Eugene code or in a Header File. Here the Device is composed of eight Parts (ordered from 5′ to 3′). This syntax includes the Device type along with the name (for readability) but the type is optional (see Supplemental Information for alternate syntax).

Device BBa_K112809(

Promoter BBa_I0500,
ORF BBa_K112805,
ORF BBa_K112806,
Terminator BBa_B0010,
Terminator BBa_B0012,
Promoter BBa_J23116,
ORF BBa_K112807,
Terminator BBa_B0010

);

#### Step 3: Instantiate the Part(s)

This entails specifying the Property values of the Part(s). This can be done in the main body of the code or in the Header File. In this case, it will be inside of *PartDeclaration.h*. For brevity, we only show the sixth of the eight parts in the Device. All eight Parts will have to be specified. Alternate syntax without explicitly assigning values to Properties exists as well (see Supplemental Information).

Promoter BBa_J23116(.ID("BBa_J23116"),

.Sequence("GATCTttgacagctagctcagtcctagggactatgctagcG"),

.Orientation("Forward"));

#### Step 4: Declare the Part(s)

Parts are collections of Properties. This again can be captured in Header Files (e.g. in *PartDefinition.h*) or in the main body. Here we show all four Part types in the design. It is the job of the designer to decide which Properties make up the individual parts. Notice the “Promoter” Part has a Property “Inducible” which can remain unspecified in Step 3.

Part Promoter(ID, Sequence, Orientation, Inducible);

Part ORF(ID, Sequence, Orientation, CDS);

Part RBS(ID, Sequence, Orientation);

Part Terminator(ID, Sequence, Orientation, Strength);

#### Step 5: Declare the Properties

Properties are text, number, or Boolean values (either arrays or single values). These represent biological characteristics associated with the design. They can be manually specified or pulled from repositories (more in the Supplemental Information).

**Figure 4:**
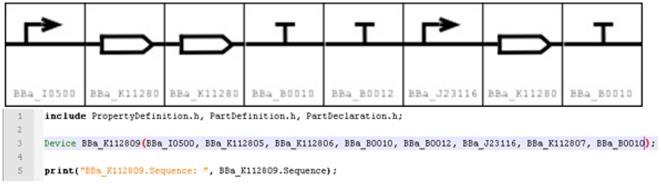
We illustrate both visually (SBOL visual; http://www.sbolstandard.org) and textually (Eugene code) an example Device (*BBa_K112809)* from the MIT registry of standardized parts. Key to notice is the fact that the three included header files encapsulate much of the design effort leaving a single line to produce the composite Device.

Property ID(txt);

Property Sequence(txt);

Property Orientation(txt);

Property CDS(boolean);

Property Strength(num[]);

Property Inducible(boolean);

The final design for *BBa_K112809* is shown in Figure 4.

Ten experimentally created Devices representative of MIT's Registry of Standard Biological Parts were created to explore the process of specifying Devices using Eugene. Table 1 in file Appendix S1 captures this exploration. Specific information on these Devices and the Eugene code for their designs can be found in the Supplemental Information.

The purpose of this exercise was to display the significance in the separation of Part and lower level Property information, which is hidden in the Header Files, from the Device level construction in the main Eugene file. As a result of this separation, an average of **85%** less code is utilized in the main file. At the same time, the ratio of DNA base pairs to total lines of code **(an average of 139∶1)** implies the portability of very complex designs to other tools or systems. Sharing designs becomes much easier, since the creation of an underlying data structure and programming interface is achieved automatically when Eugene designs are interpreted. The design interpretation times are very reasonable **(average of 95.2 ms)**. We have confidence that as designs move to encompass tens or hundreds of devices, the interpretation time will remain very reasonable.

## Results

### Design Space Exploration

The Methods section illustrates how to specify Devices with Eugene. This is only one very limited aspect of Eugene. *Design Space Exploration (DSE)* is Eugene's primary task. DSE in this context consists of two phases:

1. **Design Expansion** – This phase creates new Devices with Eugene's *permute* function. Permute goes through each of the individual Parts making up a Device and creates new Devices with other Part instances of the same type as the original Device.
2. **Design Pruning** – This phase systematically reduces the Devices created with Eugene via the application of Rules. Rules specify desired Device compositions or Part Properties. Rules and *permute* can be combined to prevent certain permutations.

**Figure 5:**
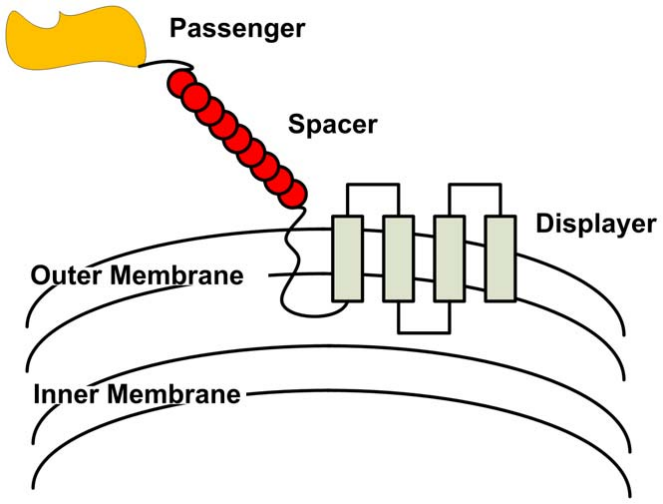
Illustration of the “cell surface display” Device case study. Here are shown the three Part types (passengers, spaces, and displayers) which when combined into a Device made up the systems that we explored. As shown the displayer interacts with the outer membrane of the bacterial cell to display the passenger protein extracellularly. Table 2 in file Appendix S1 provides more information on this system.

A *cell surface display* system built by the UC Berkeley Wetlab 2009 iGEM team went through the DSE process. This cell surface display system exposes various peptides or proteins to the extracellular environment by anchoring them to the outer membrane of *E.Coli*. The genetic Device for such a system is composed of three categories of protein domains: *passenger domains*, *displayer domains*, and *structural spacer elements*. An example of such a Device is shown in Figure 5. The individual Parts for this Device are explained briefly in Table 2 in the file Appendix S1.

### Design Expansion

There are two types of cell surface display Devices (more details in the Supplemental Information):

//A passenger/spacer/displayer/terminator Device

Device DeviceType1 (PassNeedle, SpacerINP, Disp_upaG, T01);

//Permute this device to switch out each Part instance

permute(DeviceType1);

//A passenger/displayer/terminator Device

**Figure 6:**
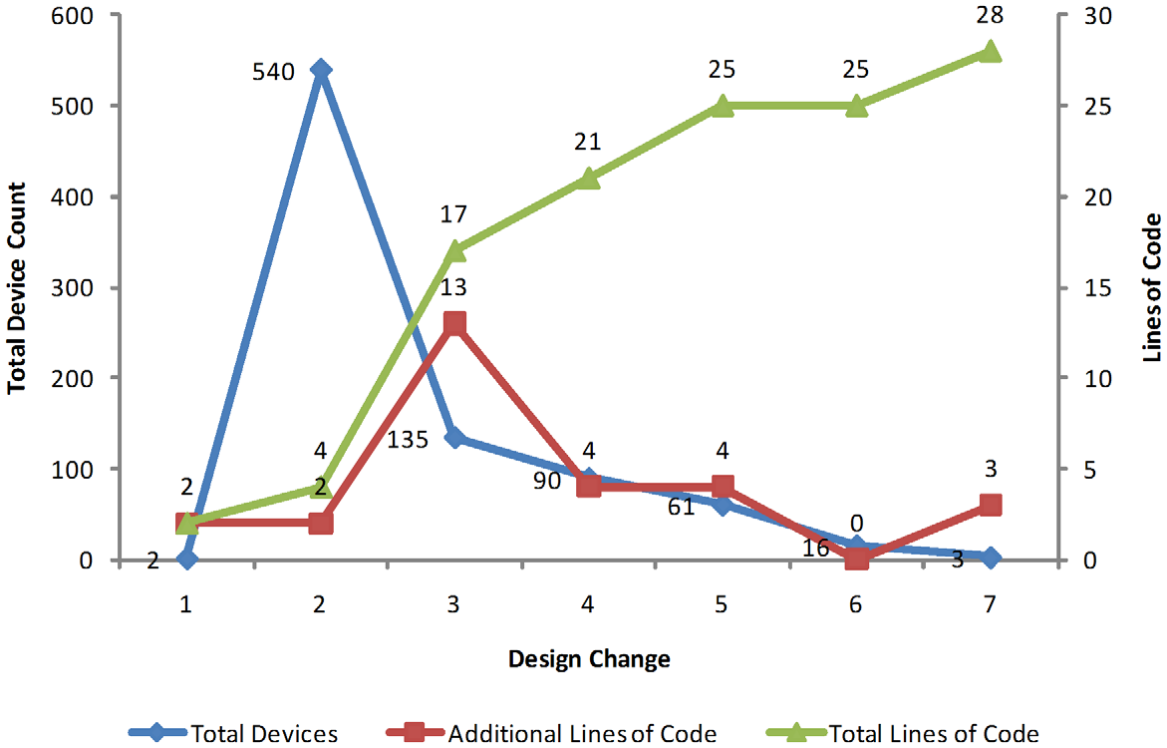
Device exploration and pruning capabilities with Eugene. This graph shows how the number of Devices created with Eugene can change with the addition of rule statements. The change in many cases can be quite dramatic with relatively few lines of code (new rules). For example with just two lines of code the initial design space explodes from two devices to 540. Then with 13 additional lines, it drops to 135 Devices. Finally, a design space of three Devices can be achieved by a total addition of 28 lines of code while still maintaining the original information to specify 540 Devices.

Device DeviceType2 (PassNeedle, Disp_upaG, T01);

permute(DeviceType2);

These four lines of code generate **540 Devices** created from the basic Parts specified initially in Eugene. Figure 6 illustrates both how our initial design space consisted of these two devices created with two lines of code, as well as the increase to 540 Devices with the addition of two permute functions (four lines total).

### Design Pruning

**Figure 7:**
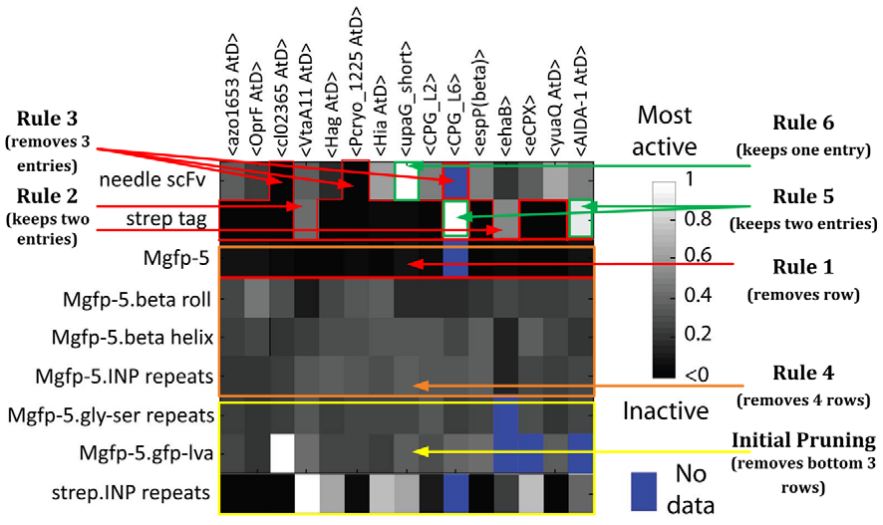
A heat map depicting the functionality of the cell surface display Devices, where the white constructs had the highest signal of functionality. This data was used to determine which Devices could be considered functional and which were not. This analysis helped to drive the development of the Eugene code. The overlaid annotations reflect a reduced heat map. This shows how Devices can be removed with the targeted application of Eugene rules. The entire design space of 90 Devices is a reduction from the original heat map's 135 Devices. Each area is labeled with the rules that affect the creation of these Devices. Rules 1-4 deal with the removal of Devices while Rules 5–6 preserve the final three highly active Devices. The x-axis is displayer domain parts and the y-axis is protein/spacer combinations.

Figure 7 is a *heat map* showing the results of assaying cell surface display Devices for functionality depending on the type of passenger used in the Device. The quantitative data sets from these assays were normalized to an appropriate control and can be used to analyze the functionality of each combination of passenger, displayer, and spacer element.

In order to reduce the design space from the original 540 Devices to the **135 Devices** in Figure 7, we added an additional **13 lines of code** (Figure 6) composed of Rule statements. The full list of these statements is in the Supplemental Information and a sample is provided here:

//Rules forbidding Ag4, Leucine Zipper, and Cellulase passengers

Rule NoAg4(NOTCONTAINS PassAg4); //Removes 90 Devices

Rule NoLeu(NOTCONTAINS PassLeu); //Removes 90 Devices

Rule NoCell(NOTCONTAINS PassCell); //Removes 90 Devices

//Rules forbidding certain passenger/spacer combos

//Do this for all 5 spacers; removes 75 devices

Rule NeedleSpacers1-5(PassNeedle NOTWITH SpacerY);

//Do this for all spacers but INP repeats; removes 60 devices

Rule StrepSpacers1–4(PassStrep NOTWITH SpacerY);

Assert(NoAg4 AND NoLeu AND NoCell AND NeedleSpacers1–4 AND StrepSpacers1–4);

We next reduced these Devices to six sets of fifteen Devices (**90 total**). These sets were combinations of three types of passengers, fifteen displayers, and three spacers. This reduction required the following **four lines**:

//Removes 45 Devices in 4 lines

Rule MgfpSpacers1(PassMgfp NOTWITH SpacerGly-Ser);

Rule MgfpSpacers2(PassMgfp NOTWITH SpacerGfp-Iva);

Rule StrepSpacers5(PassStrep NOTWITH SpacerINP);

Assert(MgfpSpacers1 AND MgfpSpacers2 AND StrepSpacers5);

To remove the inactive Devices (dark areas on Figure 7), we add the following **four lines** to reduce the space to **61 Devices**:

Rule Rule1((PassMgfp WITH SpacerBeta-Roll) OR (PassMgfp WITH SpacerBeta-Helix) OR (PassMgfp WITH SpacerINP)); //Removes 15 Devices

Rule Rule2((PassStrep WITH Disp_Vta) OR (PassStrep WITH Disp_ehaB) OR (PassStrep WITH Disp_CPG6) OR (PassStrep WITH Disp_AIDA)); //Removes 11 Devices

Rule Rule3((PassNeedle NOTWITH Disp_cl) AND (PassNeedle NOTWITH Disp_Pcryo) (PassNeedle NOTWITH Disp_CPG6)); //Removes 3 Devices

Assert(Rule1 AND Rule2 AND Rule3);

To prune the entire lower half of Figure 7, only one line is required. There are no highly active Devices in this region. This removes the need for Rule 1 for a net gain of **0 lines of code** and leaves **16 Devices**.

Rule Rule4(NOTCONTAINS PassMgfp);

Finally, to reduce the design space to only the **3 most active Devices**, we add **3 lines of code**.

//Removes all but the last 3 Devices

Rule Rule5((PassStrep WITH Disp_CPG6) OR (PassStrep WITH Disp_AIDA));

Rule Rule6(PassNeedle WITH Disp_upaG);

Assert (Rule5 AND Rule6);

With more data on these Parts, such as molecular weight, shape, efficiency, and data relevant to surface displayers, we could create more informative Properties. This would lead to more detailed, powerful rules in the future. These rules would allow more specific pruning of the combinatorial space, and the ease and specificity of the reduction would be greater still.

### Assembly and Simulation Integration

**Figure 8:**
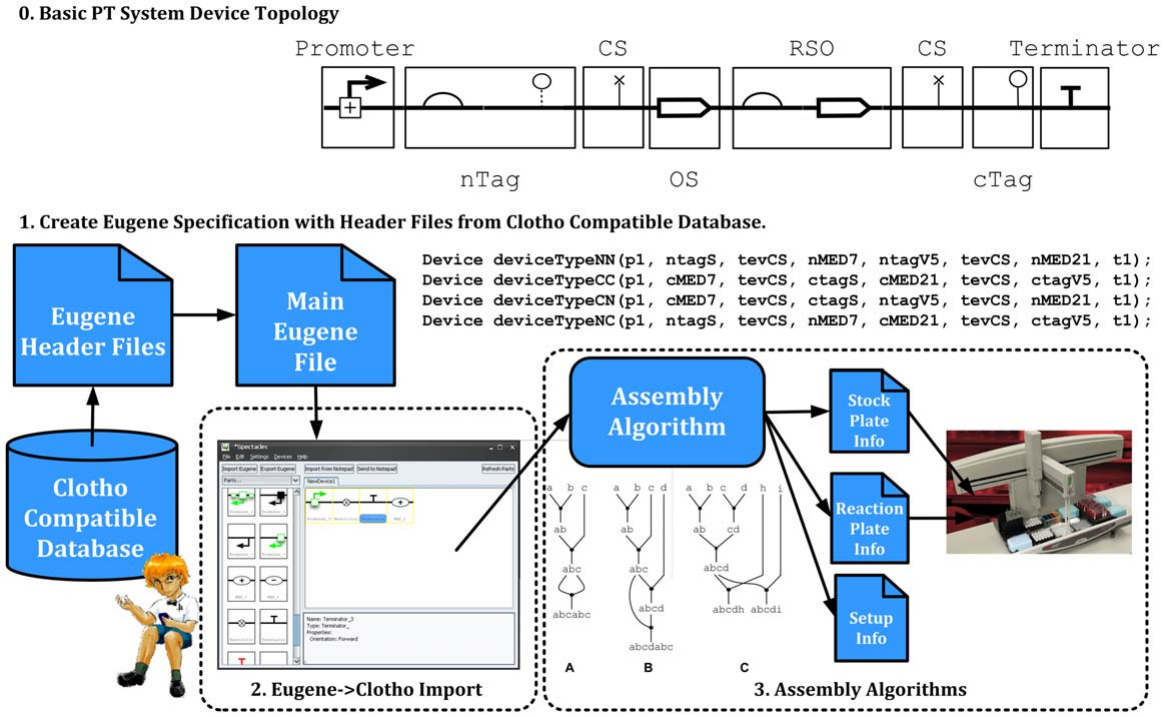
Illustration of an automated assembly flow beginning with a Eugene file for a protein tagging (PT) Device with nTag and cTag Parts. This shows the eight Parts that make up the Device and the order in which the Parts must be assembled to have a functional Device. In the Eugene import process, the Devices of interest are captured with Eugene and processed by a Clotho App (e.g. Spectacles). Later the Device construction is planned for a specific assembly protocol with the creation of an assembly graph. In the final phase, the files for a liquid handling robot are created and fed to the platform doing the assembly.

As shown, Eugene ultimately produces collections of Devices which both adhere to specific constraints and encapsulate Parts and Properties. There are two natural next steps in the design process:

1. **Automated Assembly** – 1) Determine an optimal global assembly strategy for all Devices [20]. 2) Create assembly files for a liquid handling robotic platform [21].Figure 8 illustrates this design flow. This was carried out with the help of Clotho [22], [23], (http://www.clothocad.org).
2. **Simulation** – Convert the underlying Eugene *data structures* to an exchange format for external simulation programs. We illustrate this process with the Synthetic Biology Software Suite (SynBioSS) [24].

### Automated Assembly

We created a “protein tagging” (PT) system which uses combinatorial tagging of ORFs to optimize protein expression and purification, and test protein-protein interactions, by quickly creating iterations of functional designs. Our PT systems consisted of the components types in Table 3 in file Appendix S1.

Devices were created so that each Device would encode two different ORFs where each was tagged with a different tag, either on the N- or C-terminus of the ORF. Tags were always separated from ORFs by a protease cleavage site (such that tags and ORFs can be physically separated from each other). Thus, each ORF-tag combo is made of three basic parts (one ORF, one tag, and one cleavage site between them). Therefore, a two ORF-tag architecture contains six basic parts. Since proper protein expression of a Device also requires a promoter and a terminator, each Device consists of eight basic parts in total (the six above, plus a promoter, plus a terminator). In all cases, the first Part is always a promoter, and the last Part is always a terminator. The order of the six middle Parts varies according to the desired topology of the ORF-tag combos. Figure 8 shows an example PT Device topology and sample Eugene code follows:

//Topology 1: Two nTag configuration

Device deviceTypeNN(P, nTag, CS, OS, nTag, CS, OS, T);

//Topology 2: Two cTag configuration

Device deviceTypeCC(P, RSO, CS, cTag, RSO, CS, cTag, T);

//Topology 3: cTag then nTag configuration

Device deviceTypeCN(P, RSO, CS, cTag, nTag, CS, OS, T);

//Topology 4: nTag then cTag configuration

Device deviceTypeNC(P, nTag, CS, OS, RS0, CS, cTag, T);

These four Device types result in **2304 Devices** using Eugene's permute function. We next use Rules to prevent the same antibody type of nTag or cTag from appearing in a Device. These Rules take three forms (where X is the specific tag antibody from the 12 different Part choices):

//These rules prevent specific tag combinations

Rule r1a(ctagX NOTWITH ntagX); //for CN and NC type Devices

Rule r1b(ctagX NOTMORETHAN once); //for CC type Devices

Rule r1c(ntagX NOTMORETHAN once); //for NN type Devices

This reduces the number of Devices to **2112 Devices**. We were only interested in Devices with distinct protein-tag set combinations. This is a total of **528 Devices**. See the Supplemental Information for the complete Eugene code.

Automated assembly for Eugene based Devices occurs as follows (Figure 8):

1. Create Device specifications in Eugene using Header Files created by a Clotho compatible database.
2. Use a Clotho App (e.g. Spectacles [25] or Eugene Scripter) to read in the Eugene code.
3. Clotho assembly algorithms [20] produce files for liquid handling robot based on information provided by the Clotho connection to the database (e.g. well location, sample volume, etc).

The assembly was carried out in 3 separate rounds (or stages) of assembly. In stage 1, we used 31 basic Parts to assemble 56 composite Parts (made of 2 basic Parts each). In stage 2, we used the Parts made in stage 1 to assemble 48 composite Parts (made of 4 basic Parts each). In the final stage, we used the Parts made in stage 2 to assemble 528 composite Parts (made of 8 basic Parts each). All 528 bi-cistronic operons contained a total of 3696 junctions between parts, out of which 632 were unique. Assuming $3 a Part junction and an amortized time of 10 minutes per part junction, we estimate that this saved around **$9000** ($11,088-$1,896 = $9,192) and **500 hrs** (36,960 min-6,320 min = 510 hrs). This is considering that the 528 constructs made contained a total of 3696 junctions between Parts, but of those only 632 were made since unique junctions only need to be made once.

### Simulation

**Figure 9:**
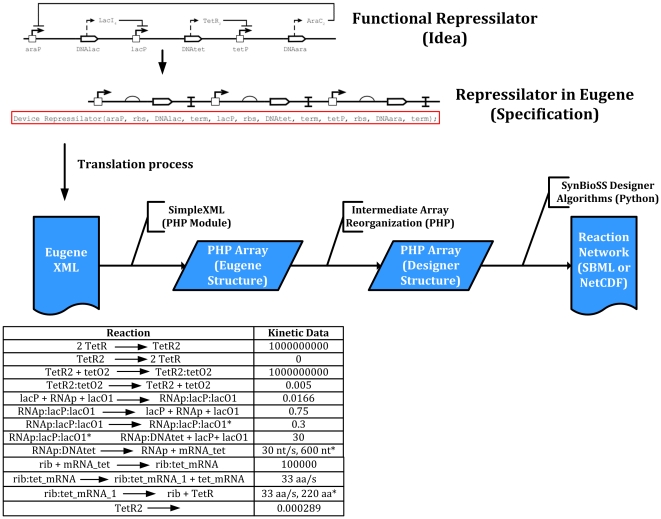
High-level diagram of a repressilator as well as its Eugene implementation. Here the relationship between LacI, TetR, and AraC and the promoters in the system is shown. This design was chosen since its behavior is well understood and can be easily decomposed into the individual Parts that make up the Device. The SynBioSS design flow with Eugene is also shown. Beginning with the Eugene XML produced by the Eugene interpreter, SimpleXML creates an array which holds the data from Eugene. After a reorganization process the data can now be transformed by SynBioSS into a reaction network in SBML or NetCDF which can then be simulated. Sample of the reaction network generated by SynBioSS Designer is also provided. These reactions describe the unregulated expression of TetR, as well as its dimerization and degradation. All rate laws are elementary and all kinetic data is in SI units unless otherwise noted. Asterisks indicate gamma-distributed reactions.

For simulation, we chose to look at a classic genetic regulatory network, namely a “repressilator” [26]. The example repressilator used here is based on a lac-tet-ara oscillatory network examined by Tuttle et al [27]. The overall behavior is that LacI represses the expression of TetR, which represses the expression of AraC, which in turn represses expression of LacI. See Figure 9 for an illustration of a repressilator. We decided to examine a repressilator because its behavior is well understood and it can be composed of primitive parts. It also provides a point of comparison with other tools in the literature (e.g. GEC [28]).

SynBioSS is a software suite for the generation, storage, and quantitative simulation of synthetic biological networks. One component of this software suite, called SynBioSS Designer, uses biological rules to create a reaction network given a series of biological parts, such as promoters and ribosome binding sites, and the spatial and temporal connectivity of these parts [29]. This reaction network represents the transcription, translation, and regulation occurring in the system. SynBioSS Designer outputs this reaction network as either a NetCDF or SBML file to be used in simulation software of the user's choice. **We use SynBioSS for this investigation but Eugene could be used with a variety of simulation tools** (e.g. Tinkercell [30]).

The Eugene code for this design is provided in the Supplemental Information. We provide a small sample here to give the reader a feel for some key elements of the repressilator design.

The following Property definitions form the pool of parameters to be associated with Parts in the repressilator:

Property Sequence(txt); //The DNA sequence for the part

Property Neg35StartEnd(txt); //Promoter information

Property Neg10StartEnd(txt); //Promoter information

Property OperatorSites(txt[]); //An array of promoter information

Property Corresponding Protein(txt); //Which protein the part produces

Property ProteinBindingInfo(txt); //Protein interaction information

The following Part definitions form the set of Part types in the repressilator and the Properties associated with them:

Part Promoter(Sequence, Neg35StartEnd, Neg10StartEnd, OperatorSites, OperatorSiteLocations);

Part RBS(Sequence);

Part CodingDNA(Sequence, CorrespondingProtein, ProteinBindingInfo);

Part Terminator(Sequence);

The following example Part declarations specify the actual physical Parts in the repressilator:

Promoter araP(); //lacI and tetR promoters created as well

RBS rbs1(); //two other RBS created as well

CodingDNA DNAlac(); //tetR and araC ORFs created as well

Terminator term1();

The following rules constrain Devices to use Parts in such a way to give rise to the repressilator behavior:

Rule promoterToCoding1(araP BEFORE DNAlac);

Rule promoterToCoding2(lacP BEFORE DNAtet);

Rule promoterToCoding3(tetP BEFORE DNAara);

Assert(promoterToCoding1 AND promoterToCoding2 AND promoterToCoding3);

Finally, the repressilator Device is declared with the specific ordering of these Parts:

Device Repressilator(araP, rbs1, DNAlac, term1, lacP, rbs2, DNAtet, term2, tetP, rbs3, DNAara, term3);

SynBioSS Designer loads this Eugene code for simulation. Specifically, Designer uses SimpleXML to load the XML produced as an artifact of Eugene interpretation. SimpleXML is a PHP extension which converts XML to an array with the same structure as the original XML. This array is then manipulated to have a structure compatible with all of Designer's algorithms. A diagram of this design flow is shown in Figure 9.

## Discussion

Eugene is a language in development. We have illustrated a very brief snapshot of its capabilities. Here are future directions for the language:

*Control Flow Extensions* – It will be important to incorporate other control statements into Eugene. The language will require the ability to systematically iterate through lists, which can be achieved through loops. This will be useful when different combinations of Parts or Devices need to be traversed and some operations on them performed.

*Functional Extensibility* - The user should have the ability to create custom functions as well. This mechanism could resemble other imperative programming languages. This process would introduce the importance of *scope* in variables and instances, since functions should only apply to specific scoped instances of variables. Currently, all variable instances in a file can be accessed globally.

*Explicit Database Support* - Another potential strength in a language like Eugene is the direct access to a database of Parts. By providing an explicit function to connect to a specified database, we would certainly give more expressional power to the language. Currently, database access is performed outside of Eugene by translating XML information from the database to Eugene code.

*Abstraction Level* – Currently, the highest level in the design hierarchy is the “Device Level”. Ideally, we would like to extend Eugene to contain Systems and the ability to operate on such a level by providing built-in functions, which will depend on new assembly standards.

*Constraint Scope* – Currently, rules are based on Part *instances* but not Part *definitions*. For example, a rule will be based on Promoter P1 but not across all Promoters. In many cases, it would be much more appropriate to apply rules to Part definitions to not only save on programming effort but also increase the expressiveness of the constraint system.

*Constraint Application* – Currently, rules are applied to Device composition. However, if one wanted to make a rule regarding two Devices, this is currently not possible. The introduction of a “System” level of abstraction with System level wide rules could address this.

We also are aware that there are a number of existing languages and tools in this domain. In particular, we consider comparisons to Systems Biology Markup Language (SBML) [31], Antimony [32], GenoCAD [33], Genetic Engineering of living Cells (GEC) [28], Proto [34], Tinkercell [30],and CellML [35] particularly relevant. In the Supplemental Information we address these comparisons directly. Broadly speaking, we feel Eugene offers certain advantages in the areas of flexibility, ease of use, interoperability with other tools, reflection of synthetic biology design flows, and extensibility.

### Summary

We have introduced the Eugene programming language for synthetic biology. In particular, we have illustrated flexible Part and Device specification and composition, combinatorial design space exploration of Devices using an expressive system of Rules, and interaction with other tools for simulation and automated assembly. We have also provided ample Supplemental Materials with comparisons to other approaches, additional information regarding our results, a complete set Eugene designs, and more information regarding how to write Eugene programs.

### Availability

Eugene is available at http://www.eugenecad.org . This is an open source project covered broadly under a BSD general license. The download includes all the examples provided here along with documentation regarding how to use the tool. In addition the grammar file used to create Eugene is available as well. It requires Java 6 (http://java.sun.com/javase/6) to run. We encourage questions and comments.

Eugene is most effectively used with other tools as illustrated in this paper. Clotho is available at http://www.clothocad.org . It too is an open source project under BSD. We highly recommend Notepad++ for the creation of Eugene files and we provide a Notepad++ syntax highlighter with the Eugene download. You can get Notepad++ at http://sourceforge.net/projects/notepad-plus/. SynBioSS is available at http://synbioss.sourceforge.net .

## Supporting Information

Appendix S1 (DOC)

## Acknowledgments

We thank the following people for making this work possible: the UC Berkeley 2009 computational iGEM team (Joanna Chen, Richard Mar, Thien Nguyen, and Nina Revko), the UC Berkeley 2009 experimental iGEM team (Jenn Brophy, Susan Chen, Elicia Farrar, Gabriela Guzman, Patrick Harrigan, Tom Huffaker, Terry Johnson, Joseph Silo, Matthew Walters, John Wang and Lane Weaver), Josh Kittleson, Tim Hsiau, the SBOL Visual team (Cesar Rodriguez, Suzie Bartram, Anusuya Ramasubramanian, Drew Endy), the JBEI Registry Team (Nathan Hillson, Tim Ham, Zinovii Dmytriv), and the CIDAR Team (Traci Haddock, Roza Ghamari in particular). In addition, the following people provided valuable input during development of Eugene: Paul Hilfinger (consulted on initial Eugene design), Jacob Beal and Ron Weiss.

## References

[1] Endy D (2005). Foundations for engineering biology.. Nature.

[2] Lucks JB, Qi L, Whitaker WR, Arkin AP (2008). Toward scalable parts families for predictable design of
biological circuits.. Curr Opin Microbiol.

[3] Arkin A (2008). Setting the standard in synthetic biology.. Nat Biotechnol.

[4] Purnick PE, Weiss R (2009). The second wave of synthetic biology: from modules to
systems.. Nat Rev Mol Cell Biol.

[5] Stevenson D (1981). A Proposed Standard for Binary Floating-Point
Arithmetic.. Computer.

[6] Bickford JH, Nassar S (1998). Handbook of bolts and bolted joints..

[7] Karwowski W (2006). Handbook on standards and guidelines in ergonomics and human
factors..

[8] Canton B, Labno A, Endy D (2008). Refinement and standardization of synthetic biological parts and
devices.. Nat Biotechnol.

[9] Gardner TS, Cantor CR, Collins JJ (2000). Construction of a genetic toggle switch in Escherichia
coli.. Nature.

[10] (2009). What's in a name?. Nature Biotechnology.

[11] Smolke CD (2009). Building outside of the box: iGEM and the BioBricks
Foundation.. Nat Biotechnol.

[12] Scott ML (2009). Programming language pragmatics..

[13] Keutzer K, Malik S, Newton AR, Rabaey JM, Sangiovanni-Vincentelli A (2000). System-level design: Orthogonalization of concerns and
platform-based design.. Ieee Transactions on Computer-Aided Design of Integrated Circuits and
Systems.

[14] Sangiovanni-Vincentelli A (2007). Quo vadis, SLD? Reasoning about the trends and challenges of
system level design.. Proceedings of the Ieee.

[15] Palnitkar S (2003). Verilog HDL: a guide to digital design and
synthesis..

[16] Ashenden PJ (2008). The designer's guide to VHDL..

[17] Chinnery D, Keutzer KW (2002). Closing the gap between ASIC & custom: tools and techniques
for high-performance ASIC design..

[18] Densmore  D, Kittleson JT, Bilitchenko L,  Liu  A, Anderson JC Rule based constraints for the construction of genetic
devices..

[19] Bilitchenko L, Liu A, Densmore D (2011). The Eugene Language for Synthetic Biology V0.03b User's
Manual and Examples.. Methods in Enzymology.

[20] Densmore D, Hsiau TH, Kittleson JT, DeLoache W, Batten C Algorithms for automated DNA assembly.. Nucleic Acids Res.

[21] Leguia M, Brophy J, Densmore D, Anderson JC (2011). Automated assembly of standard biological parts.. Methods in Enzymology.

[22] Densmore D, Devender AV, Johnson M, Sritanyaratana N (2009). A platform-based design environment for synthetic biological
systems..

[23] Bhatia S, Xia B, Bubenheim B, Dadgar M, Douglas D (2011). Clotho: A Software Platform for the Creation of Synthetic
Biological Systems A Developer's and User's Guide for Clotho
v2.. Methods in Enzymology.

[24] Hill AD, Tomshine JR, Weeding EM, Sotiropoulos V, Kaznessis YN (2008). SynBioSS: the synthetic biology modeling suite.. Bioinformatics.

[25] UC Berkeley 2009 iGEM Software Team

[26] Elowitz MB, Leibler S (2000). A synthetic oscillatory network of transcriptional
regulators.. Nature.

[27] Tuttle LM, Salis H, Tomshine J, Kaznessis YN (2005). Model-driven designs of an oscillating gene
network.. Biophys J.

[28] Pedersen M, Phillips A (2009). Towards programming languages for genetic engineering of living
cells.. J R Soc Interface.

[29] Weeding E, Houle J, Kaznessis YN (2010). SynBioSS designer: a web-based tool for the automated generation
of kinetic models for synthetic biological constructs.. Briefings in Bioinformatics.

[30] Chandran D, Bergmann FT, Sauro HM (2009). TinkerCell: modular CAD tool for synthetic
biology.. J Biol Eng.

[31] Hucka M, Finney A, Sauro HM, Bolouri H, Doyle JC (2003). The systems biology markup language (SBML): a medium for
representation and exchange of biochemical network models.. Bioinformatics.

[32] Smith LP, Bergmann FT, Chandran D, Sauro HM (2009). Antimony: a modular model definition language.. Bioinformatics.

[33] Czar MJ, Cai Y, Peccoud J (2009). Writing DNA with GenoCAD.. Nucleic Acids Res.

[34] Beal J, Bachrach J (2008). Cells Are Plausible Targets for High-Level Spatial
Languages..

[35] Lloyd CM, Halstead MD, Nielsen PF (2004). CellML: its future, present and past.. Prog Biophys Mol Biol.

