# Supplementary material for: Eugene – A Domain Specific Language for Specifying and Constraining Synthetic Biological Parts, Devices, and Systems
Source: PLoS One. 2011 Apr 29;6(4):e18882. doi: 10.1371/journal.pone.0018882 (PMC3084710; doi:10.1371/journal.pone.0018882)
Supplement: Appendix S1 — (DOC) [file pone.0018882.s001.doc]

**Supplemental Data**

The following sections provide supplementary information not contained in the main text of the paper. Much of this material provides more details on the experiments that were conducted and the Eugene code to carry them out. This code and data is also available in the Eugene distribution at: <http://sourceforge.net/projects/eugene/> or additional information at [http://www.eugenecad.org](http://www.eugenecad.org/). For questions, please contact the corresponding author of the main paper. Please note that Eugene is an evolving language. We will make every possible effort to keep Eugene backward compatible. For reference however, this code was tested with Eugene distribution v0.03b.

**Discussion of Related Work**

*Systems Biology Markup Language (SBML)* [1] is a computer-readable language used to represent models of biological processes. It is applied to the simulation of metabolism, biochemical reactions, gene regulation, and other processes where biological entities are involved and modified.  SBML allows models to be encoded in XML. Although different software tools can directly communicate and store the same representations [2] and there are numerous software tools providing a graphical interface [3], a human writable script representing biological constructs, such as biological parts or protein coding sequences, cannot be created for several reasons. First, there is no direct relationship between the syntax and the standardized Parts created by the synthetic biology community. Second, the complex syntax of XML prevents simple and brief textual descriptions. Eugene is designed to be used very differently from SBML and therefore explicitly addresses these two points while not focusing on SBML’s strengths.

*CellML* [4] is also based on XML. Its purpose is to store and exchange biological models [5] by including information about the model structure, the mathematics describing the processes, and the metadata. CellML, combined with FieldML [6] a declarative language for building hierarchical models, can describe biological information at different levels. Yet these languages cannot express the abstraction level which is reflected through the standardization effort in the synthetic biology community through the specification of Parts and Devices. Also the use of XML syntax prevents raw CellML descriptions from being human readable and limits the expression of models to visual tools only. Again, Eugene is aimed at a very different design process and therefore a direct comparison is not entirely appropriate.

*Antimony* [7] is a modular model definition language that allows scientists to define and use reaction networks. It is designed to be human-writable, unlike the previously described languages, and acts as an extension to other tools by translating the model to SBML. The notion of rules is addressed in Antimony similarly as in SMBL in the form of mathematical rules.  However, the concept of the relationship between Parts in a Device, an ordered composite of one or more Parts, cannot be expressed in such rules since Antimony and similar languages address the simulation and computational aspects of the design.

*GenoCAD* [8] is a web-based visual application guiding users through the design of Part-based genetic systems and derives the construct DNA sequence from its libraries of Parts. Rules for the construction of Parts are incorporated into the tool via a *context free grammar* [9] and validation based on the predefined *production rules* can be performed. GenoCAD researchers have also examined genetic design space exploration similarly to what we propose here [10]. Eugene and GenoCAD are more comparable than the previous tools. The fundamental difference currently is Eugene’s presence as an executable specification and interpreted language and GenoCAD’s development as a web interface supporting *attribute grammars*. GenoCAD cannot prune the design space of proposed devices based on part attributes either. Both have plans to interact with the larger community through SBOL (www.sbolstandard.org).

*Genetic Engineering of living Cells (GEC)* [11] allows logical interactions between potentially undetermined proteins and genes to be expressed in a modular manner. It also allows for the simulation of these designs with the *Systems Biology Workbench* [12] and can be translated into SBML for use with other tools. GEC includes the concepts of Parts and Devices as well and allows for the specification of primitive properties on Parts. It does not however provide automatic combinatorial exploration of devices for automated assembly with liquid handling robots or contain information on the assembly format of the Parts being used. GEC has a much more developed execution semantic as compared to Eugene and in that sense it is more mature. However Eugene is part of a larger ecosystem of tools as compared to GEC which includes SBOL (www.sbolstandard.org).

*Proto* [13] is a language originally designed for spatial computing which has been recently applied to the design of amorphous medium systems (like genetic regulatory networks). Proto has been shown to be able to create a BioBrick™ [14] network starting from a high level description of system functionality. Proto however does not have any rules for system composition nor does it involve any combinatorial design space exploration capabilities. Like GEC, it has a more formalized execution semantic compared to Eugene but no ties to the physical assembly of Devices. There are plans to integrate Proto’s “BioCompilier” with Eugene style rules.

Tinkercell [15] is a tool for the simulation of synthetic biological systems. Tinkercell provides a rich graphical environment upon which individual models of bio-chemical reactions can be associated. Designers write models to associate with the visual representations. Tinkercell is also part of the SBOL community and the authors envision a scenario where Tinkercell and Eugene can interact. Tinkercell would handle the visualization and modeling aspects for simulation with Eugene providing the design space exploration and automated assembly duties.

**Eugene Tutorial**

This section provides an overview of how a Eugene file is organized and provides a short introduction to Eugene. The figure below illustrates the pieces of a Eugene file. You see that Eugene code is organized into:

1. **Header files** – encapsulate biological information. These allow for the sharing of data across labs and projects. They also remove the need for users to enter much of the low level information needed by Eugene programs. These can be extracted automatically from databases. For example:

include PropertyDefinition.h

include PartDefinition.h

include PartDeclaration.h

1. **Properties** – define the basic information that can be captured by Eugene. This information can be textural, numeric, or arrays of these types of data. There is no set list of keywords for Properties. These are user defined and therefore it is up to the programmer to write Eugene code that interprets these values as desired. For example:

Property sequence (txt);

Property strength (num);

Property toxicity (txt);

Property uniqueID(num);

1. **Part Definitions** – collections of Properties under a given identifier (e.g. name). These are used to group Properties together in a user defined way to represent biological entities of interest. For example:

Part Promoter (sequence);

Part ORF (sequence, toxicity);

1. **Part Instantiations** – these provide the actual data required by the Part definitions. This process constitutes the creation of a biological part. For example:

Promoter p (“ACTGGT…”);

Terminator t (“GGTAAC…”, 99);

1. **Devices** – an ordered list of Part instances (or other devices). The list assumes the first entry is at the 5’ end of the DNA sequence and the last entry is the 3’ end. For example:

Device d1(p,r,o,t);

1. **Rules** –the rule operators:

*BEFORE* – **p1 BEFORE p2** – p1 must appear somewhere before p2 in all Devices where one or the other is present.

*AFTER* – **p2 AFTER p1** – p2 must appear somewhere after p1 in all Devices where one or the other is present.

*WITH* – **p1 WITH p2** – p1 and p2 must be in all Devices where one or the other is present.

*NOTWITH* – **p1 NOTWITH p2** – p1 and p2 must never appear in the same Device.

*NEXTTO* – **p1 NEXTTO p2** – p1 must be immediate before or after p2.

*NOTCONTAINS* – **NOTCONTAINS p1** – p1 must not be any Device.

*CONTAINS* – **CONTAINS p1** – all Devices must have p1.

*NOTMORETHAN* – **p1 NOTMORETHAN X** – all Devices must not have more than X instances of p1.

These rules can be applied to Part instances or Device instances. Property values of Part/Device instances or primitives in relation with one Part/Device can be operators in rule declarations when using the relational operators <, <=, >, >=, !=, ==. These operators are overloaded when evaluating text and the text is compared according to alphabetical precedence.

1. **Functions –** the *permute* function automates the specification of many Devices that share the same basic structure. It generates a Device for every combination of predefined Parts, maintaining the Part type of each component in the original Device.
2. **Conditional statements -** The use of conditional execution statements breaks up the flow of execution and allows selected blocks of code to be executed. Eugene supports two kinds of if-statements to achieve this: *Rule validating if-statement and standard if-statement*. The three logical operators AND, OR, NOT can combine statements of each type but not together.


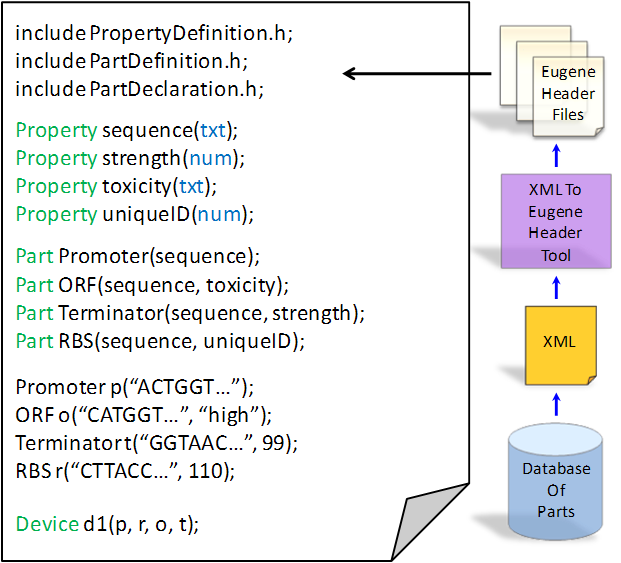


Figure 1: Overview of the organization of a Eugene file. This shows header file inclusion, Property definitions, Part definitions and instantiations, and Devices. On the right, the process of extracting information from databases to create header files automatically is outlined.


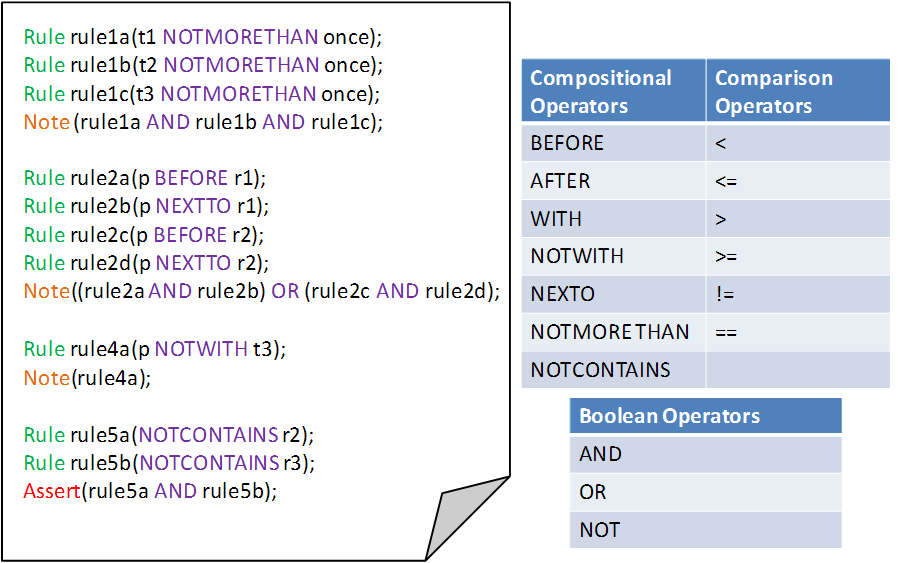


Figure 2: Rule examples are shown. Rules are compositional, comparison, or Boolean based. Rules can be created as shown and then “noted” or “asserted”. Noted rules result in warnings when rules are violated. Asserted rules prevent compilation.


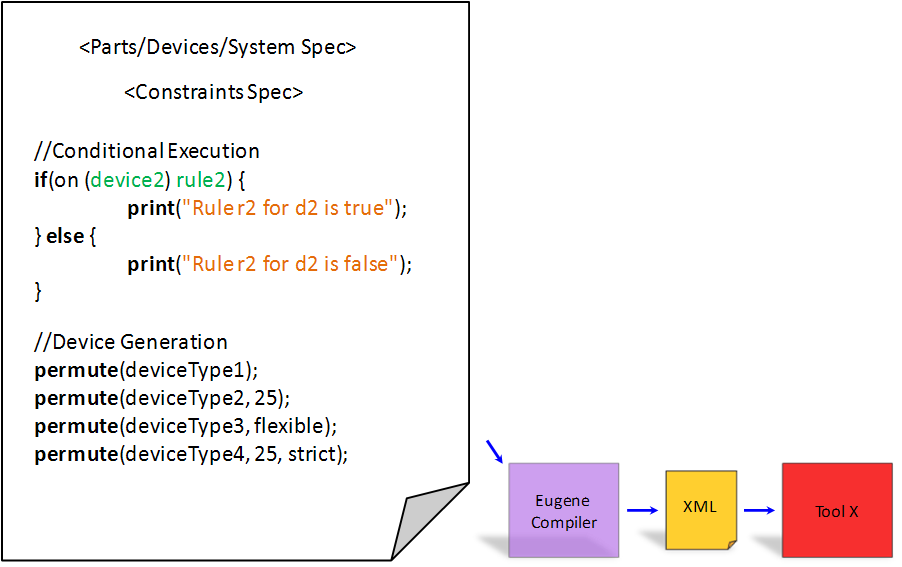


Figure 3: Eugene supports conditional execution as well as the special purpose *Permute* function. These code snippets follow the Part/Device/System specification as well as the specification of Rules. Once Eugene is “complied”, an XML description can be created which is passed to other tools (we discuss this process with SynBioSS in the main body of the paper).

/*

**Tutorial code for supplemental information**

**There are block comments**

**Property definitions can be in a header file**

**Program then needs include statement if header files are desired**

*/

//Example properties – this is a single line comment

Property BioBrickID(txt);

Property Sequence(txt);

Property someprop2(txt);

Property RelativeStrength(num);

Property someList(txt[]); //example of an array of text values

/*

Part definitions

*/

Part customP(Sequence, BioBrickID, someList);

//An example of how to add Properties to Part Definitions later

customP.**addProperties**(RelativeStrength);

txt figure = "ATCG"; // a simple text definition

/*

Part declaration/instantiation

*/

customP cp(figure, "1234",["atcg", "atc", "tcg"], 560);

//Alternate syntax; allows partial specification

customP cp2(.Sequence("GCCC"), .someList(["at", "atc", "atc"]));

num x = 50.0, y = 1000;

/*

Part definitions (again)

*/

Part Promoter(Sequence, BioBrickID, RelativeStrength);

Part RBS();

RBS.**addProperties**(Sequence);

/*

Part declarations/instantiations

One can define an instance without values in the Property labels and fill in them later

*/

RBS rs();

RBS rs2();

rs.Sequence = "GGGGGG";

rs2.Sequence = rs.Sequence;

Promoter p("GCTA", "BBa_435", 1000);

Promoter p2(.BioBrickID("ABCD"));

//Example print statement

**print**("rs2.Sequence: " , rs2.Sequence);

//Rule examples

Rule r1(p BEFORE p2);

Rule r2(cp BEFORE p);

Rule r3(p WITH p2);

Rule r4(p NEXTTO p2);

Rule r5(rs AFTER p);

Rule r6(rs NOTWITH rs2);

Rule r6a(rs WITH cp);

Rule r7(cp.Sequence != cp2.Sequence);

Rule r8(p.RelativeStrength > cp.RelativeStrength);

Rule r9(p.RelativeStrength > x);

Rule r10(y <= p.RelativeStrength);

/*

Rule r11 is wrong rule statement on purpose, error generated:

Left and right property are not of the same type.

Left property type: num and right property type: txt

*/

//Rule r11(p.RelativeStrength < p.Sequence);

/*

Rule r12 will not be evaluated and program stops without generating any errors, because >, <, >=, <= are not overloaded to evaluate strings this is in the process of being fixed

*/

//Rule r12(p.BioBrickID > p.Sequence);

/*

Assert statements will generate compile errors and terminate program

Note statements will generate warnings to the user

*/

Assert(NOT r7);

Assert(r7);

Assert((NOT r1) AND (NOT r4));

Assert(r1 AND (r2 OR r3));

Assert((r1 AND r2) OR r3);

Assert(((r1 AND r2) AND r3) OR r4 OR r5);

Assert((NOT(r1 AND r2)) OR (NOT(r2 AND r1)));

Note(NOT r7);

Note(r7);

Note((NOT r1) AND (NOT r4));

Note(r1 AND (r2 OR r3));

Note((r1 AND r2) OR r3);

Note(((r1 AND r2) AND r3) OR r4 OR r5);

Note((NOT(r1 AND r2)) OR (NOT(r2 AND r1)));

Note(r8);

Note(r9);

Note(r12);

//prints new line

**print**();

Device d0(cp);

Device d(p, cp, p2);

Device d1(p, p2, p, p);

Device d2(p2, p, p2, p, cp, p, cp, rs, p);

Device d3(cp, p, rs, p2);

Device d4(cp, cp, p, p, p2, p2, rs, p2);

Device d5(d3, rs);

Device d6(d3, d4);

Device d7(p2, p2, p2, rs);

Device d8(cp, cp, p, p, p2, rs, rs, p2);

**print**("d8.Sequence: " , d8.Sequence);

//Conditional execution examples

**if**(on (**d4**) r1) {//evaluates to true

**print**("Rule r1 on d4 is true");

txt[] a = ["a", "b", "c"], b = ["d", "d", "d"], c;

c = a + b;

**print**("a: " , a);

**print**("b: " , b);

**print**("concatenation of lists: c = a + b => ", c);

**boolean** bcheck = true;

**num** n1 = 4, n5 = 5, n6 = 6, n7 = 7;

**if**(((n1 < n5 OR n7 < n6) AND NOT bcheck) OR (n1 < n5) ) {

**print**("n1 is less n5");

} else {

**print**("n1 is greater n5");

}

**if**(on (**d2**) r2) { //evaluates to false

**print**("Rule r2 on d2 is true");

} else {

**print**("Rule r2 on d2 is not met by d2");

}

} else {

**print**("Rule r1 on d4 is not met");

txt[] a = ["a", "b", "c"], b = ["d", "d", "d"], c;

**print**("c = a + b => ", a + b);

}

//Alternate device definition syntax

Device d9(

Promoter ptry(.Sequence("1"));

Promoter ptry2(.Sequence("2"));

);

**print**("ptry.Sequence: " , ptry.Sequence);

txt newSequence = rs2.Sequence + "ATCGATCG";

**print**("rs2.Sequence: ", rs2.Sequence);

**print**("new Sequence: " , newSequence);

**MIT Parts Registry Exploration (Data)**

**Table 1: Overview of designs made with Eugene from MIT’s registry of standard biological p**arts.

| **Device** | **Group** | **Description** | **Number of** | | | **Lines of Code in Program** | | **Code Percentage Change**  **(Total to Main)** | **Compression Ratio (bp:Lines)** | **Compilation Time (ms)** |
| --- | --- | --- | --- | --- | --- | --- | --- | --- | --- | --- |
|  |  |  | Property Values | Parts | Devices | Total | Main File |  |  |  |
| BBa_K112809 | iGEM2008  UC Berkeley | T4 Lysis Device with Pbad | 21 | 7 | 0 | 19 | 3 | 84 | 158:1 | 94 |
| BBa_K112133 | iGEM2008  UC Berkeley | Xis Int temperature sensitive expression cassette | 31 | 11 | 1 | 17 | 3 | 82 | 128:1 | 94 |
| BBa_E7104 | Endy Lab | T7 consensus promoter sequence | 10 | 4 | 0 | 16 | 3 | 81 | 68:1 | 94 |
| BBa_K118021 | iGEM2008  Edingburgh | T7 consensus promoter sequence | 6 | 2 | 0 | 15 | 3 | 80 | 72:1 | 93 |
| BBa_I8510 | Knight Lab | 3OC6HSL→Inverter→ lacZalpha with orthogonal GFP protein generator | 60 | 20 | 0 | 27 | 6 | 78 | 151:1 | 109 |
| BBa_F2620 | Haseloff Lab, | 3OC6HSL → PoPS Receiver | 18 | 6 | 0 | 19 | 3 | 84 | 56:1 | 94 |
| BBa_J23040 | iGEM2006  UC Berkeley | AHL-dependent inverter | 30 | 10 | 0 | 19 | 2 | 89 | 125:1 | 94 |
| BBa_J5519 | iGEM2006  Toronto | Arabinose → LacI ts → cI 434 | 30 | 10 | 0 | 19 | 2 | 89 | 180:1 | 93 |
| BBa_J5522 | iGEM2006  Toronto | Arabinose → LacI ts → pLac → tetR →pTet | 32 | 11 | 0 | 22 | 2 | 91 | 159:1 | 93 |
| BBa_K106695 | iGEM2008  UCSF | Sir3 under a strong constitutive promoter | 8 | 4 | 0 | 16 | 2 | 88 | 291:1 | 94 |
| Total |  |  | 276 | 85 | 1 | 189 | 29 |  | 26436:189 | 952 |
| Average |  |  | 24.60 | 8.50 | 0.10 | 18.90 | 2.90 | 84.72 | 139:1 | 95.20 |

The Device ID is provided along with the research group that created it and a brief description. The number of Eugene Properties, Parts, and Devices is also shown. The statistics gathered show the lines of code total as well as how many lines appear in the main file (total lines minus header file lines). Percentage reduction of the main file code size versus the total lines is shown along with the ratio of base pairs in the Device sequence to the number of Eugene lines in the main file required to capture this info. Finally the time for interpreting the Eugene file is shown as well using a MacBook Pro at 2.4 GHz with 2 GB of memory.

Here is the information on the devices featured in Table 1s.

1. The first two Devices were created in the summer of 2008 in J.C Anderson’s lab at UC Berkeley for iGEM. These were part of the “Clonebots” project which looked at creating Devices to aid with *in-vivo* assembly techniques (gateway and self lysis in particular).
2. The third Device is from Drew Endy’s lab at Stanford University. This is a T7 consensus promoter Device.
3. The fourth Device is from Edinburgh’s 2008 iGEM team. This also is a T7 consensus promoter Device and was chosen to contrast with the previous Device from Stanford.
4. The fifth Device is from Tom Knight’s lab at MIT. This system takes 3OC6HSL as input and drives the expression of an inverter which regulates expression of lacZalpha.
5. The sixth Device is from the Haseloff lab at MIT. Again this system takes 3OC6HSL as an input to activate the LuxR transcription factor that is in turn controlled by a TetR regulated operator.
6. The seventh Device is from Berkeley’s 2006 iGEM team and is an AHL-dependent inverter.
7. The eighth and ninth parts are from Toronto’s 2006 iGEM team. The first device when induced by arabinose will produce a temperature-sensitive LacI (LacI ts) which in turn induces the production of cI 434. The second device again is induced by arabinose which ultimate leads to the production of tetR upstream of a TetR regulated promoter.
8. The tenth part is from UCSF’s 2008 iGEM which produces Sir3 under a strong constitutive promoter.

**MIT Parts Registry Exploration (Methods)**

Eugene Code: (Author: Lesia Bilitchenko)

Example BBa_E7104:

include PropertyDefinition.h, PartDefinition.h, PartDeclaration.h;

Device BBa_E7104(BBa_R0085, BBa_B0032, BBa_E0040, BBa_B0016);

print("BBa_E7104.Sequence: ", BBa_E7104.Sequence);

Example BBa_F2620:

include PropertyDefinition.h, PartDefinition.h, PartDeclaration.h;

Device BBa_F2620(BBa_R0040, BBa_B0034, BBa_C0062, BBa_B0010, BBa_B0012, BBa_R0062);

print("BBa_F2620.Sequence: ", BBa_F2620.Sequence);

Example BBa_I8510:

include PropertyDefinition.h, PartDefinition.h, PartDeclaration.h;

Rule ruleTerminators(BBa_B0010 NEXTTO BBa_B0012);

Rule rulePromoter_RBS(BBa_R0040 NEXTTO BBa_B0030);

Note (ruleTerminators AND rulePromoter_RBS);

Device BBa_I8510(

Promoter BBa_R0040,

RBS BBa_B0030,

ORF BBa_E0040,

Terminator BBa_B0010,

Terminator BBa_B0012,

Promoter BBa_R0040,

RBS BBa_B0034,

ORF BBa_C0062,

Terminator BBa_B0010,

Terminator BBa_B0012,

Promoter BBa_R0062,

RBS BBa_B0030,

ORF BBa_C2006,

Terminator BBa_B0010,

Terminator BBa_B0012,

Promoter BBa_R2000,

RBS BBa_B0034,

Reporter BBa_E0035,

Terminator BBa_B0010,

Terminator BBa_B0012,

);

if(on (BBa_I8510) ruleTerminators AND rulePromoter_RBS) {

print("BBa_I8510.Sequence: " , BBa_I8510.Sequence);

}

Example BBa_J5519:

include PropertyDefinition.h, PartDefinition.h, PartDeclaration.h;

Device BBa_J5519(BBa_I0500, BBa_B0034, BBa_J06501, BBa_B0010, BBa_B0012, BBa_R0011, BBa_B0034, BBa_C0052, BBa_B0012, BBa_B0011);

Example BBa_J5522:

include PropertyDefinition.h, PartDefinition.h, PartDeclaration.h;

Device BBa_J5522(BBa_I0500, BBa_B0034, BBa_J06501, BBa_B0010, BBa_B0012, BBa_R0011, BBa_B0033, BBa_C0040, BBa_B0010, BBa_B0012, BBa_R0040);

Example BBa_J23040:

include PropertyDefinition.h, PartDefinition.h, PartDeclaration.h;

Device BBa_J23040(BBa_R0040, BBa_B0034, BBa_C0062, BBa_B0010, BBa_B0012, BBa_R0062, BBa_B0034, BBa_C0012, BBa_B0010, BBa_B0012);

Example BBa_K106695:

include PropertyDefinition.h, PartDefinition.h, PartDeclaration.h;

Device BBa_K106695(BBa_K106694, BBa_K106692, BBa_K106017, BBa_K106018);

Example BBa_K112133:

include PropertyDefinition.h, PartDefinition.h, PartDeclaration.h;

Device BBa_K112133(BBa_K112126, BBa_K112234);

print("BBa_K112133.Sequence: ", BBa_K112133.Sequence);

Example BBa_K112809:

include PropertyDefinition.h, PartDefinition.h, PartDeclaration.h;

Device BBa_K112809(

Promoter BBa_I0500,

ORF BBa_K112805,

ORF BBa_K112806,

Terminator BBa_B0010,

Terminator BBa_B0012,

Promoter BBa_J23116,

ORF BBa_K112807,

Terminator BBa_B0010

);

print("BBa_K112809.Sequence: ", BBa_K112809.Sequence);

Example BBa_K118021:

include PropertyDefinition.h, PartDefinition.h, PartDeclaration.h;

Device BBa_K118021(

Promoter BBa_K118011,

Reporter BBa_J33204

);

print("BBa_K118021.Sequence: ", BBa_K118021.Sequence);

**Cell Surface Display Data (Info)**

**Table 2**: Cell Surface Display System

| **Part Type** | **Description** | **Part Count** |
| --- | --- | --- |
| Passenger Domain | A protein or peptide of interest exposed extracellularly not naturally found on the outermembrane of *E. Coli*. | 6 |
| Displayer Domain | Anchor for the passenger to the outer membrane The majority were autotransporters, which form pores in the outermembrane and pull their N-terminus through the pore, along with the protein attached. | 15 |
| Structural Spacer Element | A link between the passenger and displayer domains. | 5 |

**This table provides the type, description and count of the Parts which made up the *Cell Surface Display* System. In the case of the Passenger Domain, the six Parts were *Mgfp-5* (a recombinant mussel adhesive protein from *M. galloprovincialis*), a group of *Cellulases*, *Needle scFv* (a TypeIII secretion system), *streptavidin* (a strepavidin binding peptide), *Leucine zippers*, and *Ag4 peptide* (a silver binding peptide). For more information on the system see (**[**http://2009.igem.org/Team:Berkeley_Wetlab**](http://2009.igem.org/Team:Berkeley_Wetlab)**).**

**Cell Surface Display Data (Results)**

This section provides information regarding the design of “cell surface display” Devices using Eugene. We provide the Eugene code here which was used to examine the system described in the body of the main paper.

Eugene Code: (Author: Bing Xia)

//PropertyDefinition.h

Property ID(txt);

//PartDefinition.h

Part Passenger(ID);

Part Displayer(ID);

Part Spacer(ID);

Part Terminator(ID);

//PartDeclaration.h

// Terminators

Terminator T01("dblterm");

// Passengers

Passenger PassNeedle("needle scFv);

Passenger PassAg4("Ag4");

Passenger PassLeu("Leucine Zipper");

Passenger PassCell("Cellulase");

Passenger PassStrep("StregTag");

Passenger PassMgfp("Mgfp");

// Displayers

Displayer Disp_upaG("<upaG_short>");

Displayer Disp_Hia("<Hia AtD>");

Displayer Disp_yuaQ("<yuaQ AtD>");

Displayer Disp_espP("<espP(beta)>");

Displayer Disp_AIDA("<AIDA-1 AtD>");

Displayer Disp_Pcryo("<Pcryo_1225 AtD>");

Displayer Disp_cl("<cl02365 AtD>");

Displayer Disp_ehaB("<ehaB>");

Displayer Disp_Hag("<Hag AtD>");

Displayer Disp_OprF("<OprF AtD>");

Displayer Disp_CPG6("<CPG_L6>");

Displayer Disp_CPG2("<CPG_L2>");

Displayer Disp_Vta("<VtaA11 AtD>");

Displayer Disp_azo("<azo1653 AtD>");

Displayer Disp_eCPX("<eCPX>");

// Spacers

Spacer SpacerBeta-Roll("<Beta Roll>");

Spacer SpacerBeta-Helix("<Beta Helix>");

Spacer SpacerINP("<INP repeats>");

Spacer SpacerGly-Ser("(gly-ser)5");

Spacer SpacerGfp-Iva("Gfp-Iva");

//////////////// EXPLORATION ////////////////

//A passenger/spacer/displayer Device

//Can choose any specific part instance to put in initial device

Device DeviceType1 (PassNeedle, SpacerINP, Disp_upaG, T01);

//Permute this device to switch out each Part instance

permute(DeviceType1);

//A passenger/displayer Device

Device DeviceType2 (PassNeedle, Disp_upaG, T01);

permute(DeviceType2);

//////////////// PRUNING ////////////////

//Rules forbidding Ag4, Leucine, and Cellulase passengers

Rule NoAg4(NOTCONTAINS PassAg4); //Removes 90 Devices

Rule NoLeu(NOTCONTAINS PassLeu); //Removes 90 Devices

Rule NoCell(NOTCONTAINS PassCell); //Removes 90 Devices

//Rules forbidding certain passenger/spacer combos

//Do this for all 5 spacers; removes 75 devices

Rule NeedleSpacers1(PassNeedle NOTWITH SpacerBeta-Roll);

Rule NeedleSpacers2(PassNeedle NOTWITH SpacerBeta-Helix);

Rule NeedleSpacers3(PassNeedle NOTWITH SpacerINP);

Rule NeedleSpacers4(PassNeedle NOTWITH SpacerGly-Ser);

Rule NeedleSpacers5(PassNeedle NOTWITH SpacerGfp-Iva);

//Do this for all spacers but INP repeats; removes 60 devices

Rule StrepSpacers1(PassStrep NOTWITH SpacerBeta-Roll);

Rule StrepSpacers2(PassStrep NOTWITH SpacerBeta-Helix);

Rule StrepSpacers3(PassStrep NOTWITH SpacerGly-Ser);

Rule StrepSpacers4(PassStrep NOTWITH SpacerGfp-Iva);

Assert(NoAg4 AND NoLeu AND NoCell AND NeedleSpacers1 AND NeedleSpacers2 AND NeedleSpacers3 AND NeedleSpacers4 AND NeedleSpacers5 AND StrepSpacers1 AND StrepSpacers2 AND StrepSpacers2 AND StrepSpacers4);

//Removes 45 Devices in 4 lines

Rule MgfpSpacers1(PassMgfp NOTWITH SpacerGly-Ser);

Rule MgfpSpacers2(PassMgfp NOTWITH SpacerGfp-Iva);

Rule StrepSpacers5(PassStrep NOTWITH SpacerINP);

Assert(MgfpSpacers1 AND MgfpSpacers2 AND StrepSpacers5);

//Reduction to 61 functional devices

Rule Rule1((PassMgfp WITH SpacerBeta-Roll) OR (PassMgfp WITH SpacerBeta-Helix) OR (PassMgfp WITH SpacerINP)); //Removes 15 Devices

Rule Rule2((PassStrep WITH Disp_Vta) OR (PassStrep WITH Disp_ehaB) OR (PassStrep WITH Disp_CPG6) OR (PassStrep WITH Disp_AIDA)); //Removes 11 Devices

Rule Rule3((PassNeedle NOTWITH Disp_cl) AND (PassNeedle NOTWITH Disp_Pcryo) (PassNeedle NOTWITH Disp_CPG6)); //Removes 3 Devices

Assert(Rule1 AND Rule2 AND Rule3);

Rule Rule4(NOTCONTAINS PassMgfp);

//Removes all but the last 3 Devices

Rule Rule5((PassStrep WITH Disp_CPG6) OR (PassStrep WITH Disp_AIDA));

Rule Rule6(PassNeedle WITH Disp_upaG);

Assert (Rule5 AND Rule6);

**Protein Tagging Example (Info)**

**Table 3: Parts for the Protein Tagging (PT)** System

| **Part Type** | **Number** | **Description** |
| --- | --- | --- |
| Promoters (P) | 1 | “pBAD” promoter inducible using arabinose |
| Terminators (T) | 1 | Basic terminator |
| Protease Cleavage Sites (CS) | 1 | Targets a specific protease to cleave at that site. We targeted TEV sites*. |
| C-Terminus Tags (cTag) | 12 | Common epitope tags for which there are commercially available antibodies (since our assay is ELISA based): S, V5, E, HSV, VSV, T7, Strep, HA, Myc, Flag, AP, and 6XHIS. cTags were designed to tag an ORF at its C-terminus, and thus, they included a stop codon. |
| N-Terminus Tags (nTag) | 12 | Tag an ORF at its N-terminus. Include a ribosome binding site upstream of the tag start codon. RBSs were determined using the “RBS calculator algorithm” (Salis et al [16]) and the DNA sequence of each tag. |
| RBS-Start-ORF (RSO) | 2 | Specific open reading frames flanked upstream by a ribosome binding site and start codon with the omission of a downstream stop codon. |
| ORF-Stop (OS) | 2 | Specific open reading frames without a ribosome binding site but with the addition of a downstream stop codon. |

Description of the parts used in the protein tagging system. This design was used to test the automated assembly potential of Eugene. In addition to the description, the number of Parts created is also shown. This system was chosen since it was modular, created a large design space, and by design the constructs could be easily screened for functionality. * TEV is the catalytic domain of the Nuclear Inclusion a (NIa) protein encoded by the tobacco etch virus.

**Protein Tagging Example (Results)**

This section provides the code for the “protein tagging” system example provided in the main paper.

Eugene Code: (Author: Douglas Densmore)

/*

Protein Tagging Example

Author: Douglas Densmore

Date: 5/11/10

*/

//Properties for devices

Property Sequence(txt);

Property tag(txt);

//Core parts

Part Promoter(Sequence);

Part Terminator(Sequence);

Part CleavageSite(Sequence);

Part CTag(Sequence, tag);

Part NTag(Sequence, tag);

//RON and OS type parts

Part RBS_ORF_NoStop(Sequence);

Part ORF_Stop(Sequence);

//Real sequences for the promoter and terminator

Promoter p1("GATCTctatgctactccatcgagccgtcaattgtctgattcgttaccaattatgacaacttgacggctacatcattcactttttcttcacaaccggcacggaactcgctcgggctggccccggtgcattttttaaatacccgcgagaaatagagttgatcgtcaaaaccaacattgcgaccgacggtggcgataggcatccgggtggtgctcaaaagcagcttcgcctggctgatacgttggtcctcgcgccagcttaagacgctaatccctaactgctggcggaaaagatgtgacagacgcgacggcgacaagcaaacatgctgtgcgacgctggcgatatcaaaattgctgtctgccaggtgatcgctgatgtactgacaagcctcgcgtacccgattatccatcggtggatggagcgactcgttaatcgcttccatgcgccgcagtaacaattgctcaagcagatttatcgccagcagctccgaatagcgcccttccccttgcccggcgttaatgatttgcccaaacaggtcgctgaaatgcggctggtgcgcttcatccgggcgaaagaaccccgtattggcaaatattgacggccagttaagccattcatgccagtaggcgcgcggacgaaagtaaacccactggtgataccattcgcgagcctccggatgacgaccgtagtgatgaatctctcctggcgggaacagcaaaatatcacccggtcggcaaacaaattctcgtccctgatttttcaccaccccctgaccgcgaatggtgagattgagaatataacctttcattcccagcggtcggtcgataaaaaaatcgagataaccgttggcctcaatcggcgttaaacccgccaccagatgggcattaaacgagtatcccggcagcaggggatcattttgcgcttcagccatacttttcatactcccgccattcagagaagaaaccaattgtccatattgcatcagacattgccgtcactgcgtcttttactggctcttctcgctaaccaaaccggtaaccccgcttattaaaagcattctgtaacaaagcgggaccaaagccatgacaaaaacgcgtaacaaaagtgtctataatcacggcagaaaagtccacattgattatttgcacggcgtcacactttgctatgccatagcatttttatccataagattagcggatcttacctgacgctttttatcgcaactctCTACTGTTTCTCCATACCCg");

Terminator t1("GATCTccaggcatcaaataaaacgaaaggctcagtcgaaagactgggcctttcgttttatctgttgtttgtcggtgaacgctctctactagagtcacactggctcaccttcgggtgggcctttctgcgtttataG");

//Protease parts - tev

CleavageSite tevCS("GATCTGAAAACCTCTATTTTCAAGGTG");

//CTag parts (these have a stop codon)

//In addition to the sequence they also have a tag

CTag ctagS("GATCTAAAGAAACCGCTGCTGCTAAATTCGAACGTCAGCACATGGATTCCTAAG", "S");

CTag ctagV5("GATCTGGTAAGCCTATCCCTAACCCTCTGCTGGGTCTCGATTCTACGTAAG", "V5");

CTag ctagE("GATCTGGTGCTCCGGTTCCATATCCAGATCCACTGGAACCACGTTAAG", "E");

CTag ctagHSV("GATCTCAGCCGGAGCTCGCACCAGAAGACCCGGAAGACTGCtaaG", "HSV");

CTag ctagVSV("GATCTTACACCGATATAGAGATGAACAGGCTGGGAAAGtaaG", "VSV");

CTag ctagT7("GATCTATGGCTTCTATGACTGGCGGTCAGCAAATGGGTtaaG", "T7");

CTag ctagStrep("GATCTacctggagccacccgcagttcgaaaaataaG", "Strep");

CTag ctagAP("GATCTggcctgaacgatatttttgaagcgcagaaaattgaatggcatgaataaG", "AP");

CTag ctagHA("GATCTtacccatacgacgtcccagactacgctgggtaaG", "HA");

CTag ctagMYC("GATCTgaacaaaaactcatctcagaagaggatctgtaaG", "MYC");

CTag ctagFLAG("GATCTgactacaaggatgacgacgacaagtaaG", "FLAG");

CTag ctag6XHIS("GATCTCACCAtCAtCACCAtCACtaaG", "6XHIS");

//NTag parts (these will also have an RBS as well)

//In addition to the sequence they also have a tag

NTag ntagS("GATCTGAAAAGGAACCCACGCCAAGTCCAGGACACGCCATGAAAGAAACCGCTGCTGCTAAATTCGAACGTCAGCACATGGATTCCG", "S");

NTag ntagV5("GATCTGAAGCAATATCTTCACGTATAAGGAGGTATTTCATGGGTAAGCCTATCCCTAACCCTCTGCTGGGTCTCGATTCTACGG", "V5");

NTag ntagAP("GATCTCACAAACTGATACAAACGGCGGGGAAATCATGGGCCTGAACGATATTTTTGAAGCGCAGAAAATTGAATGGCATGAAG", "AP");

NTag ntagE("GATCTCACATAGCGGCGCTAAATAATAGGGAGGCGATTCAATGGGTGCTCCGGTTCCATATCCAGATCCACTGGAACCACGTG", "E");

NTag ntagHSV("GATCTCGAACACCCATATCAATATTAAAGGGAAGGTAAGCATGCAGCCGGAGCTCGCACCAGAAGACCCGGAAGACTGCG", "HSV");

NTag ntagMYC("GATCTACGCAATATCACACGGAACCGGAGGGCTAACTCGCATGTCTGAACAAAAACTCATCTCAGAAGAGGATCTGG", "MYC");

NTag ntagVSV("GATCTCAATAGAACATTAGATCAGAACGCGAAAGGGAACAATGTACACCGATATAGAGATGAACAGGCTGGGAAAGG", "VSV");

NTag ntagStrep("GATCTTACAACTACACACTATCTCATACGGAGCCCTAACATGTCTGACACCTGGAGCCACCCGCAGTTCGAAAAAG", "Strep");

NTag ntagHA("GATCTTAAGCACCCATAGAATACACACCTGGAGGAATAATGTCTTACCCATACGACGTCCCAGACTACGCTGGGG", "HA");

NTag ntagT7("GATCTGTACCAAATTAAAAAAATCAAAACGGGGTAAGATATGGCTTCTATGACTGGCGGTCAGCAAATGGGTG", "T7");

NTag ntagFLAG("GATCTGGGACAATCCCGGTCTTTAAGAACAGGAATTACATGTCTGACGATTACAAGGATGACGACGACAAGG", "FLAG");

NTag ntag6XHIS("GATCTCTACAACACCTAGCATCTAAGAAGTTACATATGGATTCTCACCATCATCACCATCACG", "6XHIS");

//Information for the 4 ORF type parts - currently they only have a sequence associated with them

//This information later can be moved to a headerfile which pulls the information from a database, it is hardcoded here for now

//Bmll4

RBS_ORF_NoStop cMED7("GATCTCAGCTAAAATCAACGTATTAAGGCACTCCATAGCGATGAAAAAATCTACCGAAAACGAATCTACCAACTACCAGTACAAAATCCAGGAACTGCGTAAACTGCTGAAATCTCTGCTGCTGAACTACCTGGAACTGATCGGTGTTCTGTCTATCAACCCGGACATGTACGAACGTAAAGTTGAAAACATCCGTACCATCCTGGTTAACATCCACCACCTGCTGAACGAATACCGTCCGCACCAGTCTCGTGAATCTCTGATCATGCTGCTGGAAGAACAGCTGGAATACAAACGTGGTGAAATCCGTGAAATCGAACAGGTTTGCAAACAGGTTCACGACAAACTGACCTCTTAAG");

//Bmll16

RBS_ORF_NoStop cMED21("GATCTGAATACCCTACTAAGGACATTCGCCAGAAAATGACCGACCGTCTGACCCAACTCCAAATTTGCCTGGACCAGATGACCGAACAGTTCTGCGCTACCCTGAACTACATCGACAAAAACCACGGTTTCGAACGTCTGACCGTTAACGAACCGCAGATGTCTGACAAACACGCTACCGTTGTTCCGCCGGAAGAGTTTTCTAACACTATTGACGAACTCTCCACCGACATCATCCTGAAAACCCGTCAGATCAACAAACTGATCGACTCTCTGCCAGGCGTTGACGTTTCCGCAGAGGAACAACTCCGTAAAATCGACATGCTCCAAAAAAAGCTCGTTGAAGTTGAAGACGAAAAAATCGAAGCTATCAAAAAAAAAGAAAAACTGCTGCGTCACGTTGACTCTCTGATCGAAGACTTCGTTGACGGTG");

//Bmll15

ORF_Stop nMED7("AAAAAATCTACCGAAAACGAATCTACCAACTACCAGTACAAAATCCAGGAACTGCGTAAACTGCTGAAATCTCTGCTGCTGAACTACCTGGAACTGATCGGTGTTCTGTCTATCAACCCGGACATGTACGAACGTAAAGTTGAAAACATCCGTACCATCCTGGTTAACATCCACCACCTGCTGAACGAATACCGTCCGCACCAGTCTCGTGAATCTCTGATCATGCTGCTGGAAGAACAGCTGGAATACAAACGTGGTGAAATCCGTGAAATCGAACAGGTTTGCAAACAGGTTCACGACAAACTGACCTCTtaa");

//Bmll17

ORF_Stop nMED21("ACCGACCGTCTGACCCAACTCCAAATTTGCCTGGACCAGATGACCGAACAGTTCTGCGCTACCCTGAACTACATCGACAAAAACCACGGTTTCGAACGTCTGACCGTTAACGAACCGCAGATGTCTGACAAACACGCTACCGTTGTTCCGCCGGAAGAGTTTTCTAACACTATTGACGAACTCTCCACCGACATCATCCTGAAAACCCGTCAGATCAACAAACTGATCGACTCTCTGCCAGGCGTTGACGTTTCCGCAGAGGAACAACTCCGTAAAATCGACATGCTCCAAAAAAAGCTCGTTGAAGTTGAAGACGAAAAAATCGAAGCTATCAAAAAAAAAGAAAAACTGCTGCGTCACGTTGACTCTCTGATCGAAGACTTCGTTGACGGTtaa");

//These rules prevent the same tag used between c and n tags

Rule r1a(ctagS NOTWITH ntagS);

Rule r2a(ctagV5 NOTWITH ntagV5);

Rule r3a(ctagAP NOTWITH ntagAP);

Rule r4a(ctagE NOTWITH ntagE);

Rule r5a(ctagHSV NOTWITH ntagHSV);

Rule r6a(ctagMYC NOTWITH ntagMYC);

Rule r7a(ctagVSV NOTWITH ntagVSV);

Rule r8a(ctagStrep NOTWITH ntagStrep);

Rule r9a(ctagHA NOTWITH ntagHA);

Rule r10a(ctagT7 NOTWITH ntagT7);

Rule r11a(ctagFLAG NOTWITH ntagFLAG);

Rule r12a(ctag6XHIS NOTWITH ntag6XHIS);

//these rules make sure that no ctag tag is used more than once

num once = 1;

Rule r1b(ctagS NOTMORETHAN once);

Rule r2b(ctagV5 NOTMORETHAN once);

Rule r3b(ctagAP NOTMORETHAN once);

Rule r4b(ctagE NOTMORETHAN once);

Rule r5b(ctagHSV NOTMORETHAN once);

Rule r6b(ctagMYC NOTMORETHAN once);

Rule r7b(ctagVSV NOTMORETHAN once);

Rule r8b(ctagStrep NOTMORETHAN once);

Rule r9b(ctagHA NOTMORETHAN once);

Rule r10b(ctagT7 NOTMORETHAN once);

Rule r11b(ctagFLAG NOTMORETHAN once);

Rule r12b(ctag6XHIS NOTMORETHAN once);

//these rules make sure that no ntag tag is used more than once

Rule r1c(ntagS NOTMORETHAN once);

Rule r2c(ntagV5 NOTMORETHAN once);

Rule r3c(ntagAP NOTMORETHAN once);

Rule r4c(ntagE NOTMORETHAN once);

Rule r5c(ntagHSV NOTMORETHAN once);

Rule r6c(ntagMYC NOTMORETHAN once);

Rule r7c(ntagVSV NOTMORETHAN once);

Rule r8c(ntagStrep NOTMORETHAN once);

Rule r9c(ntagHA NOTMORETHAN once);

Rule r10c(ntagT7 NOTMORETHAN once);

Rule r11c(ntagFLAG NOTMORETHAN once);

Rule r12c(ntag6XHIS NOTMORETHAN once);

//These notes are the way we "activate" the checking of rules

Assert(r1a);

Assert(r2a);

Assert(r3a);

Assert(r4a);

Assert(r5a);

Assert(r6a);

Assert(r7a);

Assert(r8a);

Assert(r9a);

Assert(r10a);

Assert(r11a);

Assert(r12a);

Assert(r1b);

Assert(r2b);

Assert(r3b);

Assert(r4b);

Assert(r5b);

Assert(r6b);

Assert(r7b);

Assert(r8b);

Assert(r9b);

Assert(r10b);

Assert(r11b);

Assert(r12b);

Assert(r1c);

Assert(r2c);

Assert(r3c);

Assert(r4c);

Assert(r5c);

Assert(r6c);

Assert(r7c);

Assert(r8c);

Assert(r9c);

Assert(r10c);

Assert(r11c);

Assert(r12c);

//The four basic types of devices we want to make

Device deviceTypeNN(p1, ntagS, tevCS, nMED7, ntagV5, tevCS, nMED21, t1);

Device deviceTypeCC(p1, cMED7, tevCS, ctagS, cMED21, tevCS, ctagV5, t1);

Device deviceTypeCN(p1, cMED7, tevCS, ctagS, ntagV5, tevCS, nMED21, t1);

Device deviceTypeNC(p1, ntagS, tevCS, nMED7, cMED21, tevCS, ctagV5, t1);

//These functions will make all the devices

//All set to strict; so that they check the assert statements

//Limited the number produced for clarity

permute(deviceTypeNN, 25, strict);

permute(deviceTypeCC, 25, strict);

permute(deviceTypeCN, 25, strict);

permute(deviceTypeNC, 25, strict);

**Repressilator Design (Results)**

This section details both the Eugene code and Eugene XML produced for the “respressilator” design example system.

Sequence and details of the promoters in the design:

araP: TTTACATAGCATTTTTATCCATAATATGTTAGCGGATCCTAAGC

- -35 start/end = [1,4]
- -10 start/end = [25,29]
- araI1 start/end = [5,24]
- araI2 start/end = [30,44]

lacP: TTGACATTGTGAGCGGATAACAAGATACT

- -35 start/end = [1,6]
- -10 start/end = [24,29]
- lacO1 start/end = [7,23]

tetP: TTGACA TCCCTATCAGTGATAGAGA TACT

- -35 start/end = [1,6]
- -10 start/end = [26,29]
- tetO2 start/end = [7,25]

Eugene Code: (Author: Adam Liu)

/* Property definitions */

Property Sequence(txt);

Property Neg35StartEnd(txt);

Property Neg10StartEnd(txt);

Property OperatorSites(txt[]);

Property OperatorSiteLocations(txt[]);

Property CorrespondingProtein(txt);

/* Part definitions */

Part Promoter(Sequence, Neg35StartEnd, Neg10StartEnd, OperatorSites, OperatorSiteLocations);

Part RBS(Sequence);

Part CodingDNA(Sequence, CorrespondingProtein);

Part Terminator(Sequence);

/* Part declarations */

Promoter araP(.Sequence("TTTACATAGCATTTTTATCCATAATATGTTAGCGGATCCTAAGC"), .Neg35StartEnd("[1,4]"), .Neg10StartEnd("[25,29]"), .OperatorSites(["araI1","araI2"]), .OperatorSiteLocations(["[5,24]", "[30,44]"]));

Promoter lacP(.Sequence("TTGACATTGTGAGCGGATAACAAGATACT"), .Neg35StartEnd("[1,6]"), .Neg10StartEnd("[24,29]"), .OperatorSites(["lacO1"]), .OperatorSiteLocations(["[7,23]"]));

Promoter tetP(.Sequence("TTGACATCCCTATCAGTGATAGAGATACT"), .Neg35StartEnd("[1,6]"), .Neg10StartEnd("[26,29]"), .OperatorSites(["tetO2"]), .OperatorSiteLocations(["[7,25]"]));

RBS rbs1();

RBS rbs2();

RBS rbs3();

CodingDNA DNAlac(.CorrespondingProtein("LacI"));

CodingDNA DNAtet(.CorrespondingProtein("TetR"));

CodingDNA DNAara(.CorrespondingProtein("araC"));

Terminator term1();

Terminator term2();

Terminator term3();

/* Rule declarations and assertions */

// Each promoter appears before an RBS.

Rule promoterBeforeRBS1(araP BEFORE rbs1);

Rule promoterBeforeRBS2(lacP BEFORE rbs2);

Rule promoterBeforeRBS3(tetP BEFORE rbs3);

Assert(promoterBeforeRBS1 AND promoterBeforeRBS2 AND promoterBeforeRBS3);

// Each RBS appears immediately before the coding region.

Rule rbsBeforeCoding1(rbs1 BEFORE DNAlac);

Rule rbsNextToCoding1(rbs1 NEXTTO DNAlac);

Rule rbsBeforeCoding2(rbs2 BEFORE DNAtet);

Rule rbsNextToCoding2(rbs2 NEXTTO DNAtet);

Rule rbsBeforeCoding3(rbs3 BEFORE DNAara);

Rule rbsNextToCoding3(rbs3 NEXTTO DNAara);

Assert(rbsBeforeCoding1 AND rbsNextToCoding1);

Assert(rbsBeforeCoding2 AND rbsNextToCoding2);

Assert(rbsBeforeCoding3 AND rbsNextToCoding3);

// A terminator appears immediately after the coding region.

Rule termAfterCoding1(term1 AFTER DNAlac);

Rule termNextToCoding1(term1 NEXTTO DNAlac);

Rule termAfterCoding2(term2 AFTER DNAtet);

Rule termNextToCoding2(term2 NEXTTO DNAtet);

Rule termAfterCoding3(term3 AFTER DNAara);

Rule termNextToCoding3(term3 NEXTTO DNAara);

Assert(termAfterCoding1 AND termNextToCoding1);

Assert(termAfterCoding2 AND termNextToCoding2);

Assert(termAfterCoding3 AND termNextToCoding3);

// Specific coding regions appear before the appropriate promoter.

Rule promoterToCoding1(araP BEFORE DNAlac);

Rule promoterToCoding2(lacP BEFORE DNAtet);

Rule promoterToCoding3(tetP BEFORE DNAara);

Assert(promoterToCoding1 AND promoterToCoding2 AND promoterToCoding3);

// Each promoter and gene only appears once on the device.

num x = 1;

Rule uniquePromoter1(araP NOTMORETHAN x);

Rule uniqueCoding1(DNAlac NOTMORETHAN x);

Rule uniquePromoter2(lacP NOTMORETHAN x);

Rule uniqueCoding2(DNAtet NOTMORETHAN x);

Rule uniquePromoter3(tetP NOTMORETHAN x);

Rule uniqueCoding3(DNAara NOTMORETHAN x);

Assert(uniquePromoter1 AND uniqueCoding1);

Assert(uniquePromoter2 AND uniqueCoding2);

Assert(uniquePromoter3 AND uniqueCoding3);

/* Repressilator device declaration */

Device Repressilator(araP, rbs1, DNAlac, term1, lacP, rbs2, DNAtet, term2, tetP, rbs3, DNAara, term3);

Eugene XML: (Author: Auto-generated by Eugene interpreter)

<?xml version="1.0" encoding="UTF-8" standalone="no" ?>

<design>

<parts>

<part name="**rbs3**" type="**RBS**">

<sequence />

</part>

<part name="**DNAara**" type="**CodingDNA**">

<sequence />

<correspondingprotein>**araC**</correspondingprotein>

</part>

<part name="**DNAlac**" type="**CodingDNA**">

<sequence />

<correspondingprotein>**LacI**</correspondingprotein>

</part>

<part name="**lacP**" type="**Promoter**">

<operatorsitelocations>**[[7,23]]**</operatorsitelocations>

<operatorsites>**[lacO1]**</operatorsites>

<neg35startend>**[1,6]**</neg35startend>

<sequence>**TTGACATTGTGAGCGGATAACAAGATACT**</sequence>

<neg10startend>**[24,29]**</neg10startend>

</part>

<part name="**araP**" type="**Promoter**">

<operatorsitelocations>**[[5,24], [30,44]]**</operatorsitelocations>

<operatorsites>**[araI1, araI2]**</operatorsites>

<neg35startend>**[1,4]**</neg35startend>

<sequence>**TTTACATAGCATTTTTATCCATAATATGTTAGCGGATCCTAAGC**</sequence>

<neg10startend>**[25,29]**</neg10startend>

</part>

<part name="**term1**" type="**Terminator**">

<sequence />

</part>

<part name="**term3**" type="**Terminator**">

<sequence />

</part>

<part name="**term2**" type="**Terminator**">

<sequence />

</part>

<part name="**tetP**" type="**Promoter**">

<operatorsitelocations>**[[7,25]]**</operatorsitelocations>

<operatorsites>**[tetO2]**</operatorsites>

<neg35startend>**[1,6]**</neg35startend>

<sequence>**TTGACATCCCTATCAGTGATAGAGATACT**</sequence>

<neg10startend>**[26,29]**</neg10startend>

</part>

<part name="**DNAtet**" type="**CodingDNA**">

<sequence />

<correspondingprotein>**TetR**</correspondingprotein>

</part>

<part name="**rbs2**" type="**RBS**">

<sequence />

</part>

<part name="**rbs1**" type="**RBS**">

<sequence />

</part>

</parts>

<devices>

<device name="**Repressilator**">

<components>

<component type="**Promoter**">**araP**</component>

<component type="**RBS**">**rbs1**</component>

<component type="**CodingDNA**">**DNAlac**</component>

<component type="**Terminator**">**term1**</component>

<component type="**Promoter**">**lacP**</component>

<component type="**RBS**">**rbs2**</component>

<component type="**CodingDNA**">**DNAtet**</component>

<component type="**Terminator**">**term2**</component>

<component type="**Promoter**">**tetP**</component>

<component type="**RBS**">**rbs3**</component>

<component type="**CodingDNA**">**DNAara**</component>

<component type="**Terminator**">**term3**</component>

</components>

</device>

</devices>

</design>

**References**

1. Hucka M, Finney A, Sauro HM, Bolouri H, Doyle JC, et al. (2003) The systems biology markup language (SBML): a medium for representation and exchange of biochemical network models. Bioinformatics 19: 524-531.

2. Mirschel S, Steinmetz K, Rempel M, Ginkel M, Gilles ED (2009) PROMOT: modular modeling for systems biology. Bioinformatics 25: 687-689.

3. Funahashi A. Celldesigner: A Modeling Tool for Biochemical Networks. In: Matsuoka Y, Jouraku A, Kitano H, Kikuchi N, editors; 2006. pp. 1707-1712.

4. Lloyd CM, Halstead MD, Nielsen PF (2004) CellML: its future, present and past. Prog Biophys Mol Biol 85: 433-450.

5. Cooling MT, Rouilly V, Misirli G, Lawson J, Yu T, et al. Standard virtual biological parts: a repository of modular modeling components for synthetic biology. Bioinformatics 26: 925-931.

6. Christie GR, Nielsen PM, Blackett SA, Bradley CP, Hunter PJ (2009) FieldML: concepts and implementation. Philos Transact A Math Phys Eng Sci 367: 1869-1884.

7. Smith LP, Bergmann FT, Chandran D, Sauro HM (2009) Antimony: a modular model definition language. Bioinformatics 25: 2452-2454.

8. Czar MJ, Cai Y, Peccoud J (2009) Writing DNA with GenoCAD. Nucleic Acids Res 37: W40-47.

9. Cai Y, Hartnett B, Gustafsson C, Peccoud J (2007) A syntactic model to design and verify synthetic genetic constructs derived from standard biological parts. Bioinformatics 23: 2760-2767.

10. Cai Y, Lux MW, Adam L, Peccoud J (2009) Modeling structure-function relationships in synthetic DNA sequences using attribute grammars. PLoS Comput Biol 5: e1000529.

11. Pedersen M, Phillips A (2009) Towards programming languages for genetic engineering of living cells. J R Soc Interface 6 Suppl 4: S437-450.

12. Sauro HM, Hucka M, Finney A, Wellock C, Bolouri H, et al. (2003) Next generation simulation tools: the Systems Biology Workbench and BioSPICE integration. OMICS 7: 355-372.

13. Beal J, Bachrach J (2008) Cells Are Plausible Targets for High-Level Spatial Languages. Second IEEE International Conference on Self-Adaptive and Self-Organizing Systems Workshops. pp. 284-291.

14. Shetty RP, Endy D, Knight TF, Jr. (2008) Engineering BioBrick vectors from BioBrick parts. J Biol Eng 2: 5.

15. Chandran D, Bergmann FT, Sauro HM (2009) TinkerCell: modular CAD tool for synthetic biology. J Biol Eng 3: 19.

16. Salis HM, Mirsky EA, Voigt CA (2009) Automated design of synthetic ribosome binding sites to control protein expression. Nat Biotechnol 27: 946-950.
